# Supplementary material for: Vascular Anastomoses and Dissection: A Six-Part Simulation Curriculum for Surgical Residents
Source: MedEdPORTAL. 2024 May 28;20:11406. doi: 10.15766/mep_2374-8265.11406 (PMC11219091; doi:10.15766/mep_2374-8265.11406)
Supplement: Supplementary file 1 — Session 1 - End-to-End Anastomoses.docxSession 2 - End-to-Side Anastomoses.docxSession 3 - Cadaveric Vein Anastomoses.docxSession 4 - Aortic Exposure and Anastomosis.docxSession 5 - Vein Harvest.docxSession 6 - Extremity Bypass.docxSurveys.docx [file mep_2374-8265.11406-s001.zip › D. Session 4 - Aortic Exposure and Anastomosis.docx]

**Appendix D: Session Four Details**

*Use this appendix to plan and execute the fourth session of the curriculum.*

*Pictures contained in this appendix are author owned.*

**Aortic Exposure and Anastomosis**

***Summary:*** ***This two-hour session involves dissecting out the thoracic aorta in a porcine model and performing an end-to-side anastomosis with and without a Carrel patch. Residents will focus on tissue dissection and reinforce lessons around vascular anastomosis.***

***Objectives:***

By the end of the session, residents should be able to:

- Isolate the thoracic and/or abdominal aorta with major branches from the surrounding retroperitoneal structures in a porcine model.
- Identify, divide, and transpose the celiac or subclavian artery onto the aorta using an end-to-side anastomosis.
- Identify, divide, and transpose the superior mesenteric artery (SMA) or carotid artery onto the abdominal aorta with a Carrel patch using an end-to-side anastomosis.

***Equipment:***

We use standard skills lab supplies (*) and purchased materials (^‡^) for this session. The following should be available for each pair of trainees:

- Fine needle driver (e.g., Castro or Ryder/BM27)*
- Pickups (e.g., DeBakey) x3*
- Fine pickups (e.g., Gerald or fine DeBakey) x3*
- Right angle*
- Tonsil clamps x2*
- Rubber shod x4*
- Metzenbaum scissors*
- 11 blade scalpel*
- Porcine tissue model (request heart, lung, aorta, and trachea) containing descending aorta and branches^‡^
- 5-0 polypropylene (e.g., Prolene or Surgipro) suture x4*
- 4-0 silk ties*

***Set Up:***

- Before the session, email residents with session objectives, steps, and tips/tricks. Optionally, advise them to bring Loupes if available.
- Recruit vascular surgical faculty and/or advanced trainees (e.g., fellows) to circulate during the session and provide assistance.
- Spread out tissue model (aorta and branches) on a tray or Chux (Picture 4A).
- Place materials at each well-lit station.

***Session Steps and Timeline:***

- Introduce trainees to the objectives and task steps (5 minutes).
- Expose the descending thoracic and abdominal aorta and the visceral vessels and free them from perivascular tissue, ligating small side branches using ties and larger branches with suture ligation (Picture 4B). If vessel injury occurs, repair injuries with 5-0 polypropylene sutures. Time permitting, completely excise the aorta from surrounding tissue (Picture 4C) (30 minutes).
- Divide the celiac artery or subclavian artery at its origin (depending on the tissue available). Oversew the aortic side using 5-0 prolene (10 minutes).
- Gently spatulate the celiac or subclavian artery and create an aortotomy to transpose the artery to the aorta using a scalpel and scissors (Picture 5D) (5 minutes).
- Perform an end-to-side anastomosis to re-implant the celiac or subclavian artery (35 minutes).
  - Begin the anastomosis by placing a stitch at the heel. Tie down the first stitch with three throws and shod the ends. Place a stay suture at the toe to align the anastomosis for optimal needle angle and access, and shod the ends.
  - Sew starting from the heel and proceeding along the anastomotic edge. Adjust the arteries with forceps to create the correct needle angles. Continue with this suture to the toe of the artery. Remove the stay suture and continue around the toe for three or four stitches
  - Begin sewing with the remaining heel suture and sew towards the toe until the anastomosis is completed (Picture 5E).
- Remove the SMA or carotid artery from the aorta at its orifice including at least 5mm of aorta circumferentially to create a Carrel patch using scissors. Then create an aortotomy using a scalpel and scissors in the aorta at a new site (5 minutes).
- Perform an end-to-side anastomosis to re-implant the SMA or carotid artery and Carrel patch (30 minutes).
  - Begin the anastomosis by placing a stitch at one end of the patch. Tie down the first stitch with three throws and shod the ends. Place a stay suture at the other end of the patch to align the anastomosis for optimal needle angle and access, and shod the ends.
  - Sew starting from the one end and proceeding along the circumference of the anastomosis proceeding until just over half of the anastomosis has been completed. Adjust the arteries with forceps to create the correct angles.
  - Repeat this technique for the second half of the anastomosis (Picture 5F).
- Perform a debrief with all residents to discuss challenges and lessons learned (10 minutes).

***Tips and Tricks:***

- Identify and then follow the areolar tissue plane around the aorta to isolate it from surrounding structure.
- Discourage excessive spreading during dissection; when there is clear tissue to take, it should be cut.
- Encourage tissue triangulation to expedite dissection.
- The thick wall of the aorta requires a 90-degree angle of needle entry.


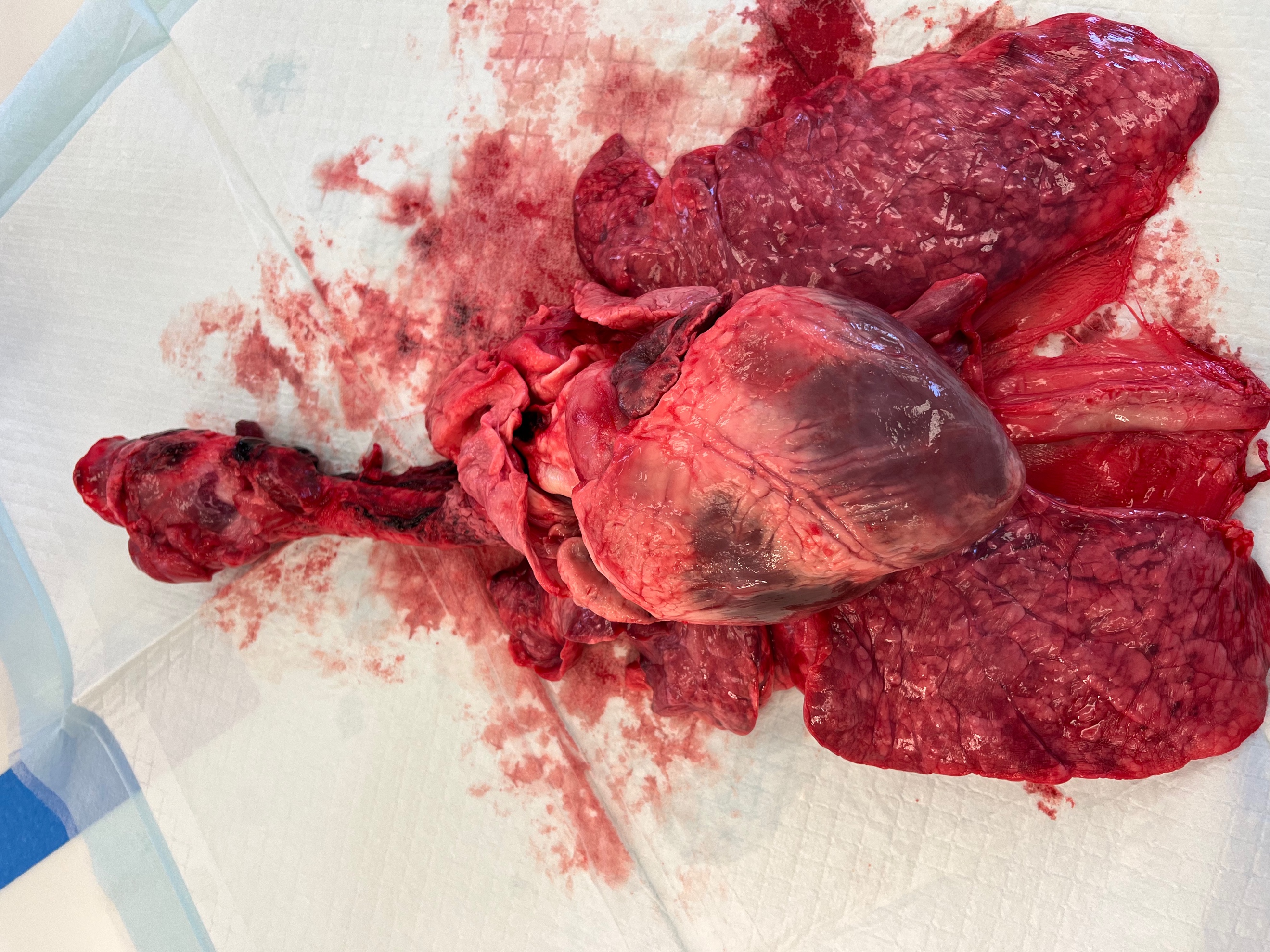


Picture 4A: Spread out the tissue model on a tray or Chux


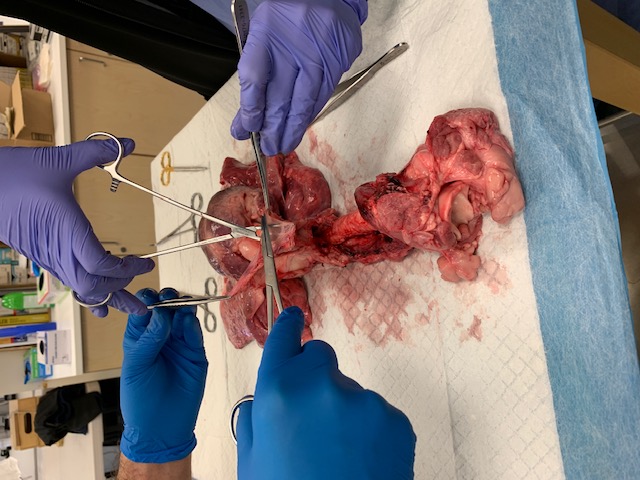


Picture 4B: Work in pairs to dissect away perivascular tissue

**
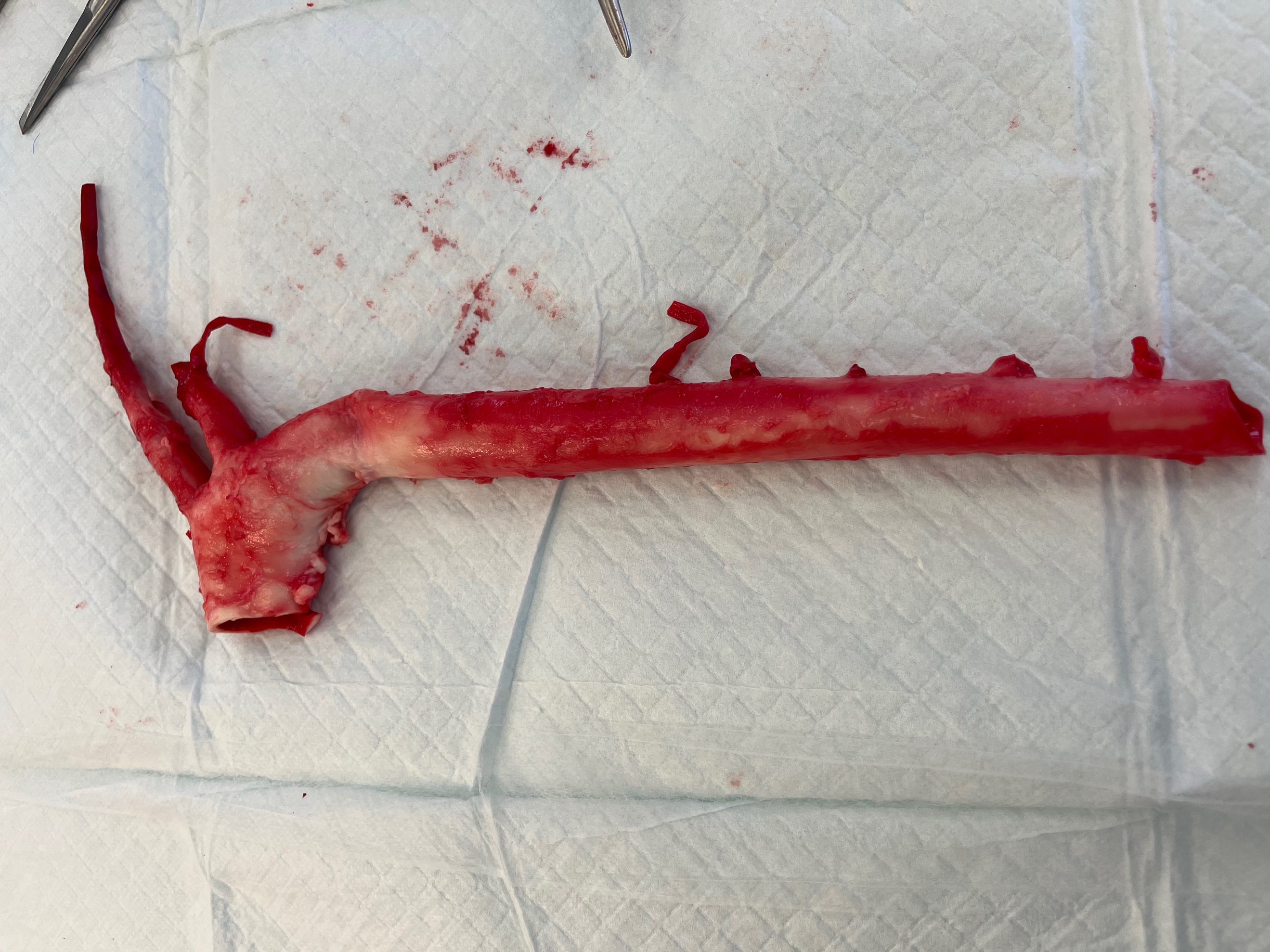
**

Picture 4C: Excised aorta

**
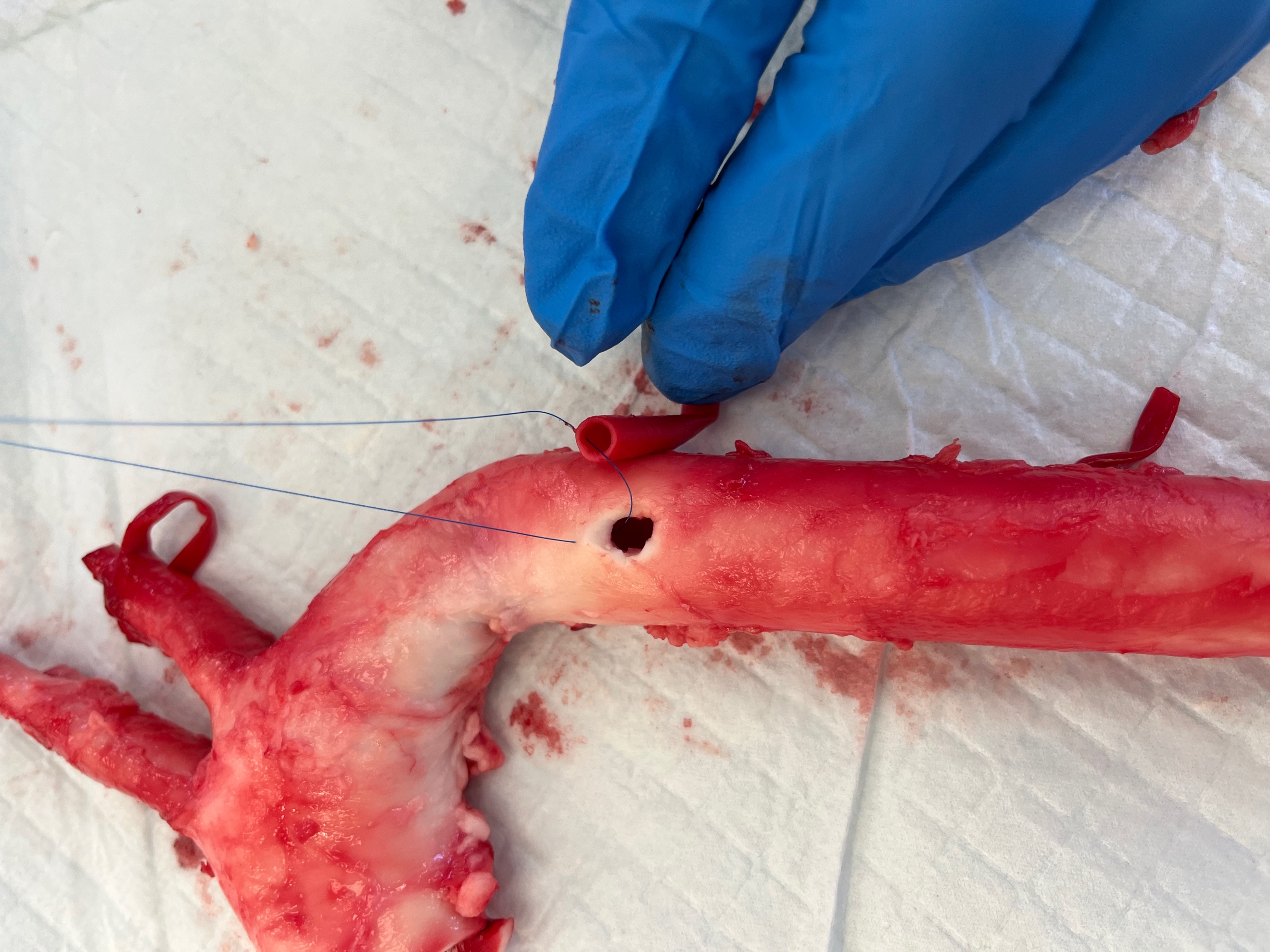
**

Picture 4D: Transpose the celiac or subclavian artery onto an aortotomy

**
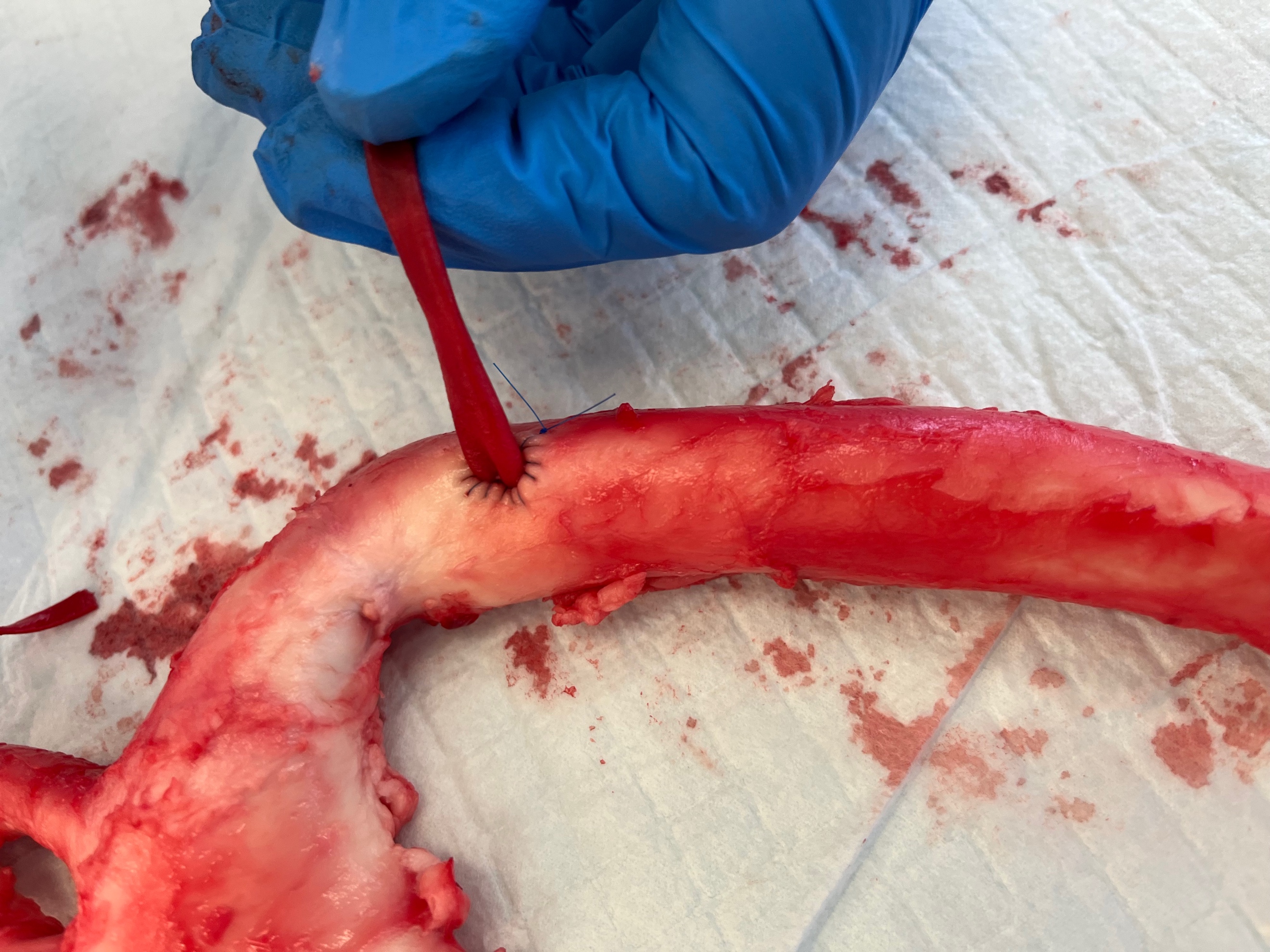
**

Picture 4E: Completed end-to-side anastomosis

**
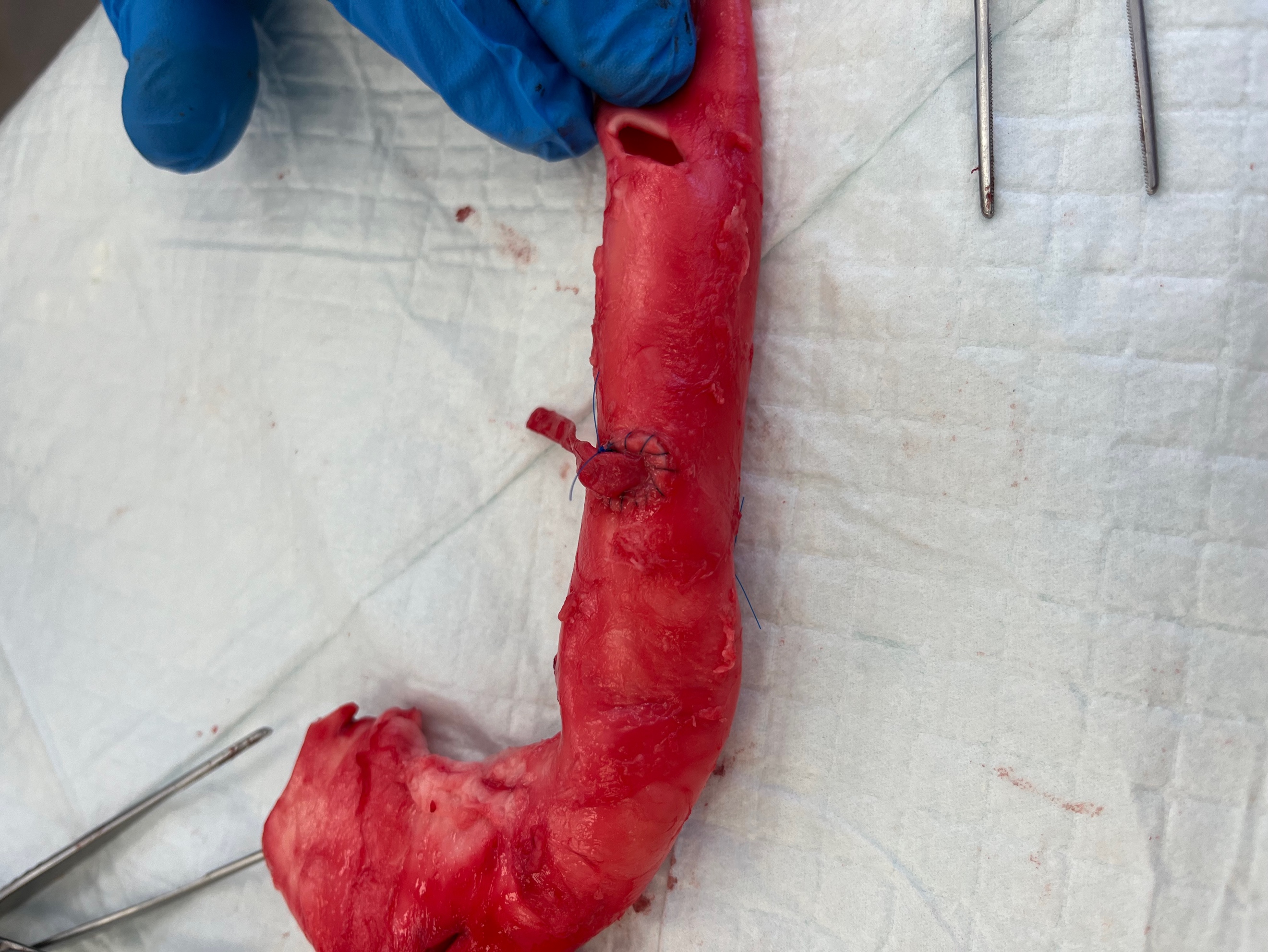
**

Picture 4F: Completed end-to-side anastomosis with Carrel patch
