# Supplementary material for: Vascular Anastomoses and Dissection: A Six-Part Simulation Curriculum for Surgical Residents
Source: MedEdPORTAL. 2024 May 28;20:11406. doi: 10.15766/mep_2374-8265.11406 (PMC11219091; doi:10.15766/mep_2374-8265.11406)
Supplement: Supplementary file 1 — Session 1 - End-to-End Anastomoses.docxSession 2 - End-to-Side Anastomoses.docxSession 3 - Cadaveric Vein Anastomoses.docxSession 4 - Aortic Exposure and Anastomosis.docxSession 5 - Vein Harvest.docxSession 6 - Extremity Bypass.docxSurveys.docx [file mep_2374-8265.11406-s001.zip › E. Session 5 - Vein Harvest.docx]

**Appendix E: Session Five Details**

*Use this appendix to plan and execute the fifth session of the curriculum.*

*Pictures contained in this appendix are author owned.*

**Vein Harvest**

***Summary:*** ***This two-hour session involves isolating a porcine splenic vein. This challenging model has similarities to the operative saphenous vein harvest.***

***Objectives:***

By the end of the session, residents should be able to:

- Harvest a length of intact splenic vein from a porcine spleen model.
- Tie off venous side branches, taking care not to compromise the lumen.
- Repair leaks as needed using 6-0 polypropylene stitches.

***Equipment:***

We use standard skills lab supplies (*) and purchased materials (^‡^) for this session. The following should be available for each pair of trainees:

- Fine needle driver (e.g., Castro or Ryder/BM27)*
- Fine pickups (e.g., Gerald or fine DeBakey) x3*
- Right angle*
- Tonsil clamps x2*
- Metzenbaum scissors*
- Porcine tissue model of spleen^‡^
- Wooden board with nails*
- 6-0 polypropylene (e.g., Prolene or Surgipro) suture x2*
- 3-0 and 4-0 silk ties*
- Ruler*
- Red dye*
- 20cc syringe with cannula*

***Set Up:***

- Before the session, email residents with session objectives, steps, and tips/tricks. Optionally, advise them to bring Loupes if available.
- Recruit vascular surgical faculty and/or advanced trainees (e.g., fellows) to circulate during the session and provide assistance.
- Spread out tissue model (porcine spleen) on a wooden board and nail it in place at three sites (Picture 5A).
- Place materials at each well-lit station.

***Session Steps and Timeline:***

- Introduce trainees to the objectives and task steps (5 minutes).
- Identify the splenic vein and, staying within the perivascular plane, expose the vein along its length (Picture 5B). Use a combination of blunt and sharp dissection to remove the fibrous bands of peri-vascular tissue, isolating and ligating vein branches along the way (Picture 5C). If vessel injury occurs, repair injuries when identified with 6-0 polypropylene sutures (100 minutes).
- Cannulate the vein and inject red dye to check for patency and leaks (Picture 5D). If there are leaks, repair them using 6-0 polypropylene stitches and then re-check for leaks (5 minutes).
- Perform a debrief with all residents to discuss challenges and lessons learned (10 minutes).

***Tips and Tricks:***

- Encourage tissue triangulation to expedite dissection.
- Note that all branches are on the splenic side of the vein
- Stay in the proper plane by dissecting right along the splenic vein.
- Tie in atraumatic fashion when ligating venous branches to prevent avulsion.
- Place ties about 2mm from the vein to avoid creating stenoses.
- Sutures can be placed around the vein to assist with retraction.

Picture 5A: Place the porcine spleen on a wooden board


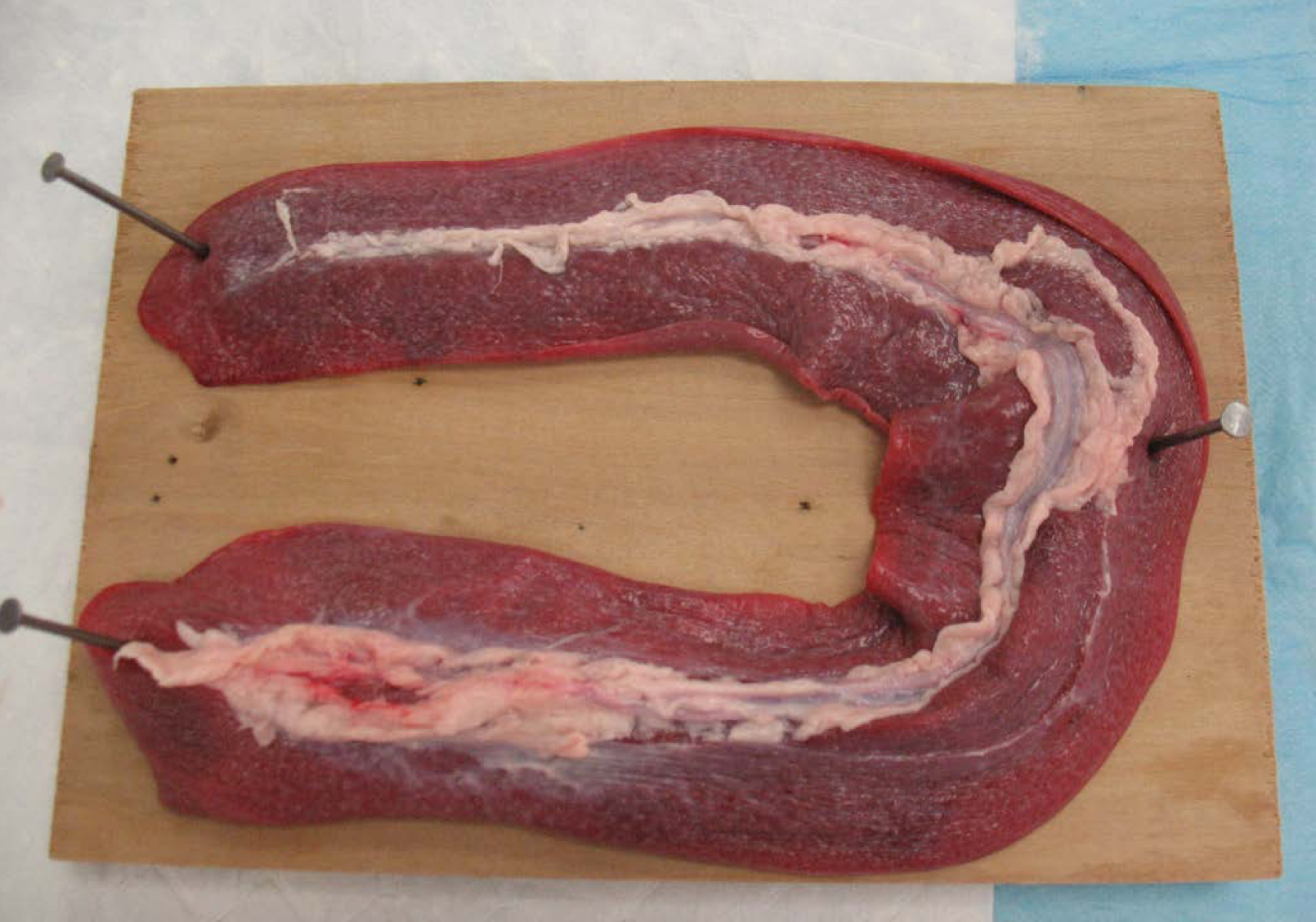


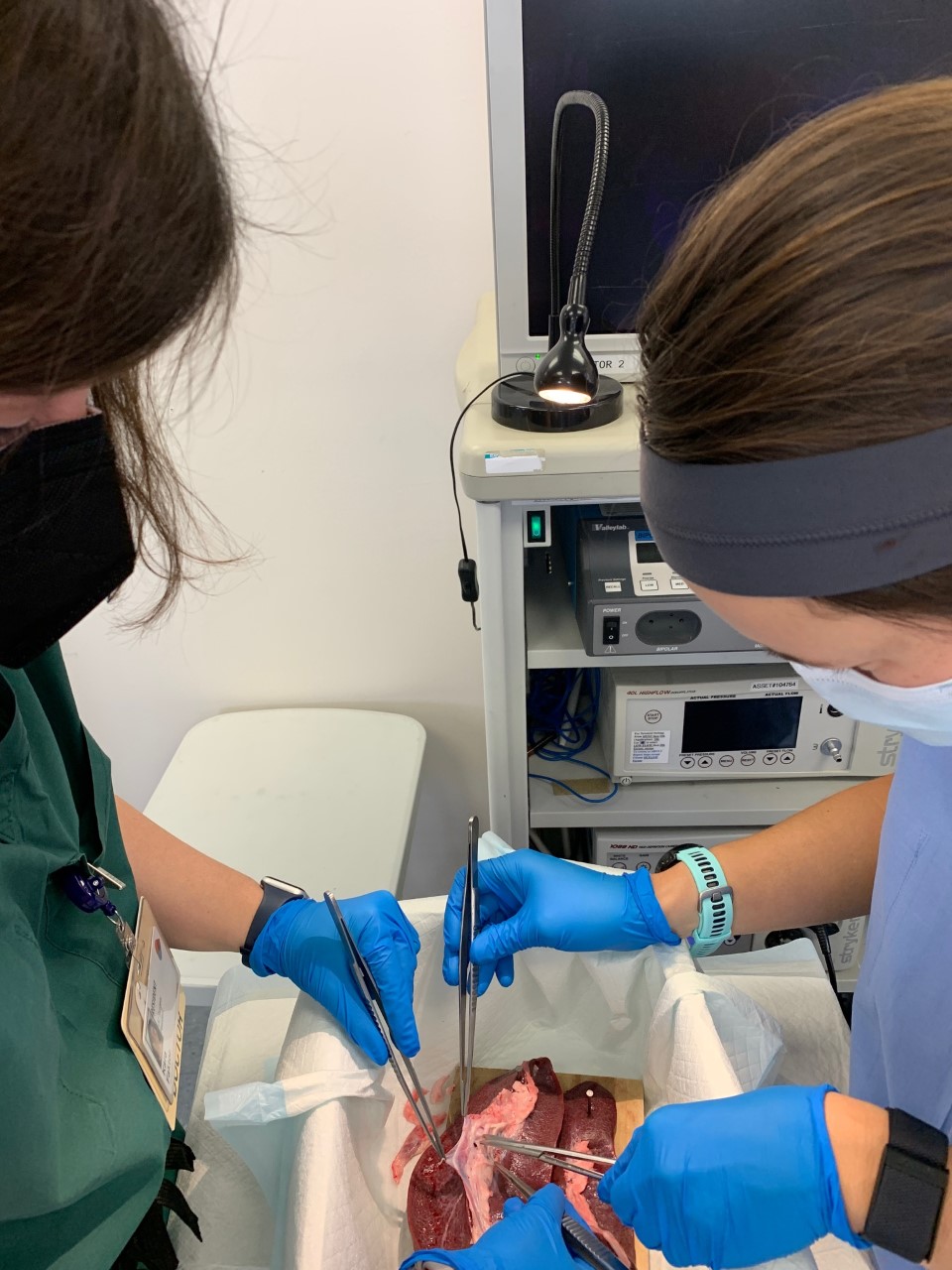


Picture 5B: Expose the vein along its length with a combination of sharp and blunt dissection

Picture 5C: Ligate side branches with silk ties


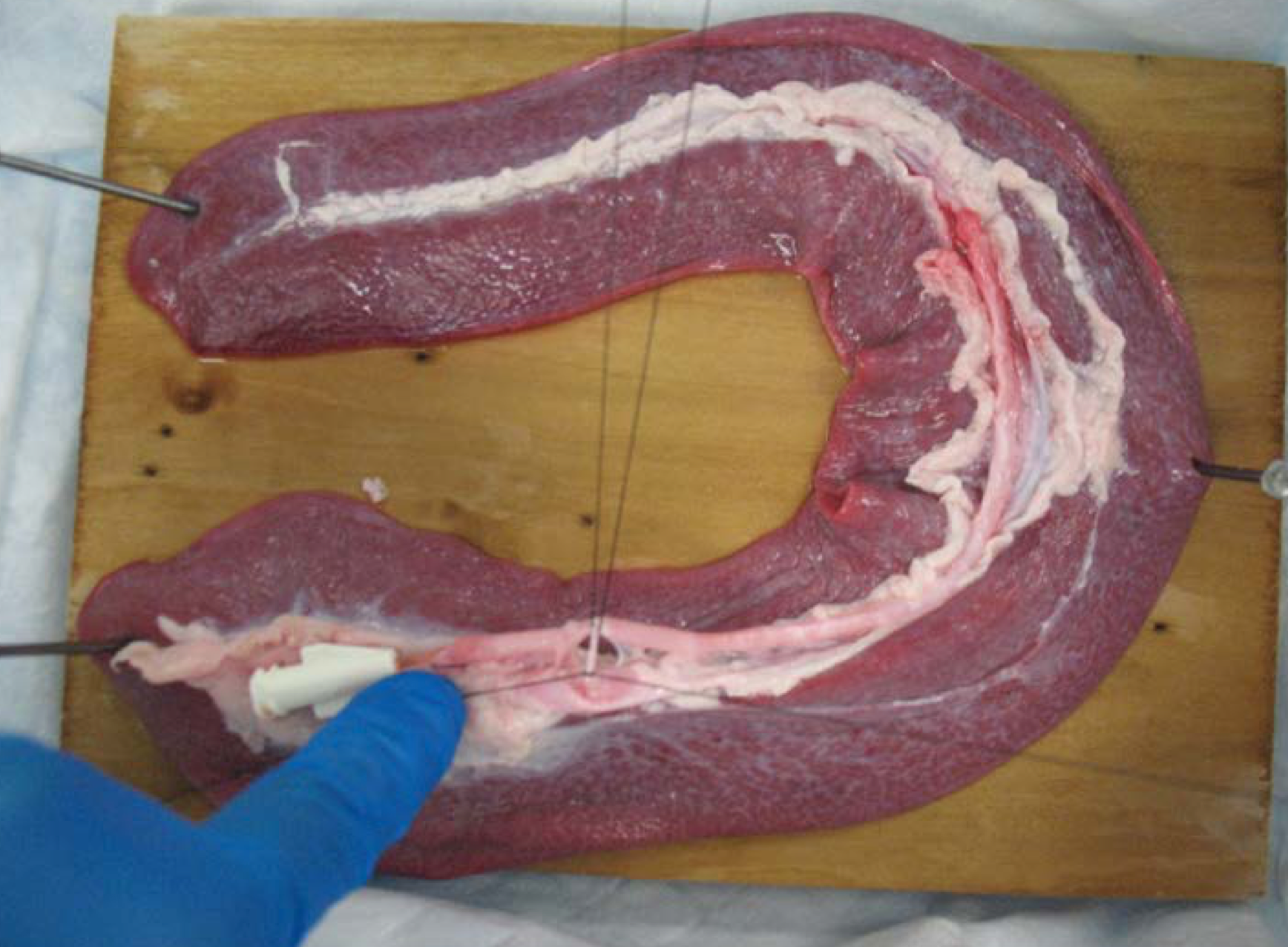


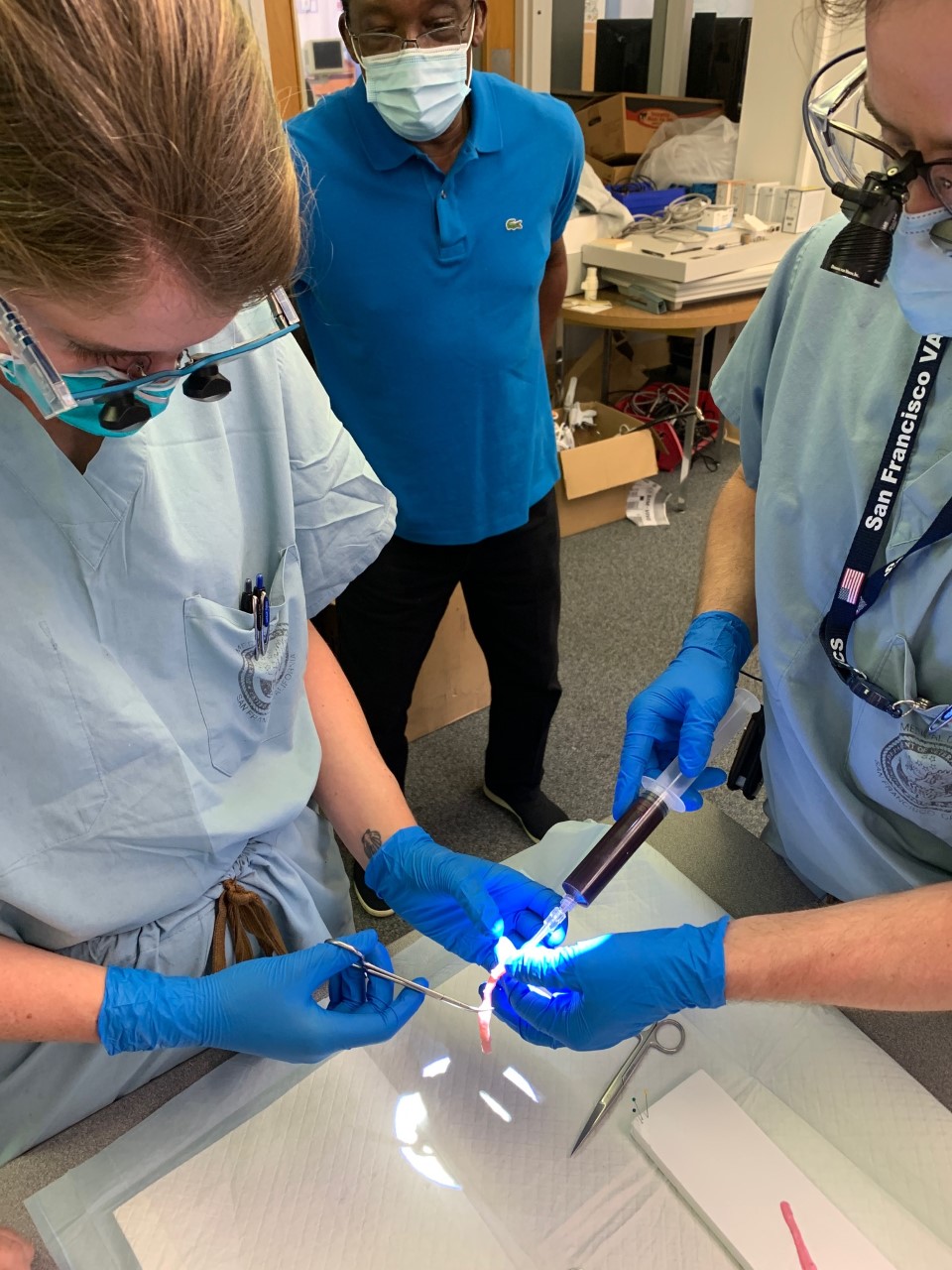


Picture 5D: Cannulate the vein and check for leaks with a red dye solution. Repair leaks with polypropylene stitches
