# Supplementary material for: Vascular Anastomoses and Dissection: A Six-Part Simulation Curriculum for Surgical Residents
Source: MedEdPORTAL. 2024 May 28;20:11406. doi: 10.15766/mep_2374-8265.11406 (PMC11219091; doi:10.15766/mep_2374-8265.11406)
Supplement: Supplementary file 1 — Session 1 - End-to-End Anastomoses.docxSession 2 - End-to-Side Anastomoses.docxSession 3 - Cadaveric Vein Anastomoses.docxSession 4 - Aortic Exposure and Anastomosis.docxSession 5 - Vein Harvest.docxSession 6 - Extremity Bypass.docxSurveys.docx [file mep_2374-8265.11406-s001.zip › C. Session 3 - Cadaveric Vein Anastomoses.docx]

**Appendix C: Session Three Details**

*Use this appendix to plan and execute the third session of the curriculum.*

*Pictures contained in this appendix are author owned.*

**Cadaveric Vein Anastomoses**

***Summary:*** ***This two-hour session involves an end-to-end anastomosis superficially and an end-to-side anastomosis at depth using cadaveric vein. Residents will continue to work on vascular suturing skills with the added challenges of controlling the more pliable cadaveric tissue.***

***Objectives:***

By the end of the session, residents should be able to:

- Join two pieces of cadaveric graft in an end-to-end fashion superficially and end-to-side fashion at depth without twisting or narrowing the graft.
- Adjust the tissue and needle with each bite to create an evenly spaced suture path without catching the back wall.
- Assist the suturing surgeon by providing tissue retraction to maintain an open and clear field of view.

***Equipment:***

We use standard skills lab supplies (*) and materials obtained through donation (^†^) for this session. The following should be available for each pair of trainees:

- Fine needle driver (e.g., Castro or Ryder/BM27)*
- Fine pickups (e.g., Gerald or fine DeBakey) x3*
- Rubber shod x4*
- Metzenbaum scissors*
- 11 blade scalpel*
- Ceramic tile*
- Suction cup clips x2*
- Cadaveric vein (8cm length, cut in half)^†^
- Plastic box with central hole cut out*
- 5-0 or 6-0 polypropylene (e.g., Prolene or Surgipro) suture x4*

***Set Up:***

- Before the session, email residents with session objectives, steps, and tips/tricks. Optionally, advise them to bring Loupes if available.
- Recruit vascular surgical faculty and/or advanced trainees (e.g., fellows) to circulate during the session and provide assistance.
- Attach suction cup clips opposite from each other on the ceramic tile.
- Place remaining materials at each well-lit station.

***Session Steps and Timeline:***

- Introduce trainees to the objectives and task steps (5 minutes).
- Cut the length of vein into two pieces, spatulating the cut end (Picture 3A). Clip each length of vein into a suction cup such that the spatulated ends meet in the middle (5 minutes).
- Begin the anastomosis by aligning the vein’s heel and toe (Figure 3B). Place stay sutures at both the heel and toe and then shod the ends (Picture 3C) (10 minutes).
- Secure one of the stay sutures with three throws and sew midway to the second stay suture. Then secure the second stay suture and complete the first half of the anastomosis, tying the stay sutures to each other (20 minutes).
  - Repeat this technique for the second half of the anastomosis (20 minutes).
  - Cannulate the vein and inject red dye to check for patency and leaks (Picture 3D). If there are leaks, repair them using 6-0 polypropylene stitches and then re-check for leaks (5 minutes).
- Time permitting, place a length of PTFE between clips and put the ceramic tile at the bottom of the box to simulate a vessel at depth.
  - Make an “arteriotomy” in the clipped piece of PTFE using the 11 blade scalpel and scissors. Spatulate the vein such that the heel-to-toe length matches the length of the “arteriotomy” (5 minutes).
  - Begin the anastomosis by placing a stitch at the heel. Tie down the first stitch with three throws and shod the ends. Alternately, a parachuting technique can be used. Place a stay suture at the toe to align the anastomosis for optimal needle angle and access, and shod the ends (10 minutes).
  - Sew starting from the heel and proceeding along the anastomotic edge. Adjust the vein with forceps to create the correct needle angles (20 minutes). Continue with this suture to the toe of the vein. Remove the stay suture and continue around the toe for three or four stitches.
    - Begin sewing with the remaining heel suture and sew towards the toe until the anastomosis is completed (15 minutes).
- Perform a debrief with all residents to discuss challenges and lessons learned (10 minutes).

***Tips and Tricks:***

- Residents have all completed each necessary step using PTFE; the added challenge in this session results from the use of the more pliable vein.
- Moisten the vein with a spray bottle during the session to prevent it from drying out.

Picture 3A: Spatulate the end of each of the two pieces of cryovein


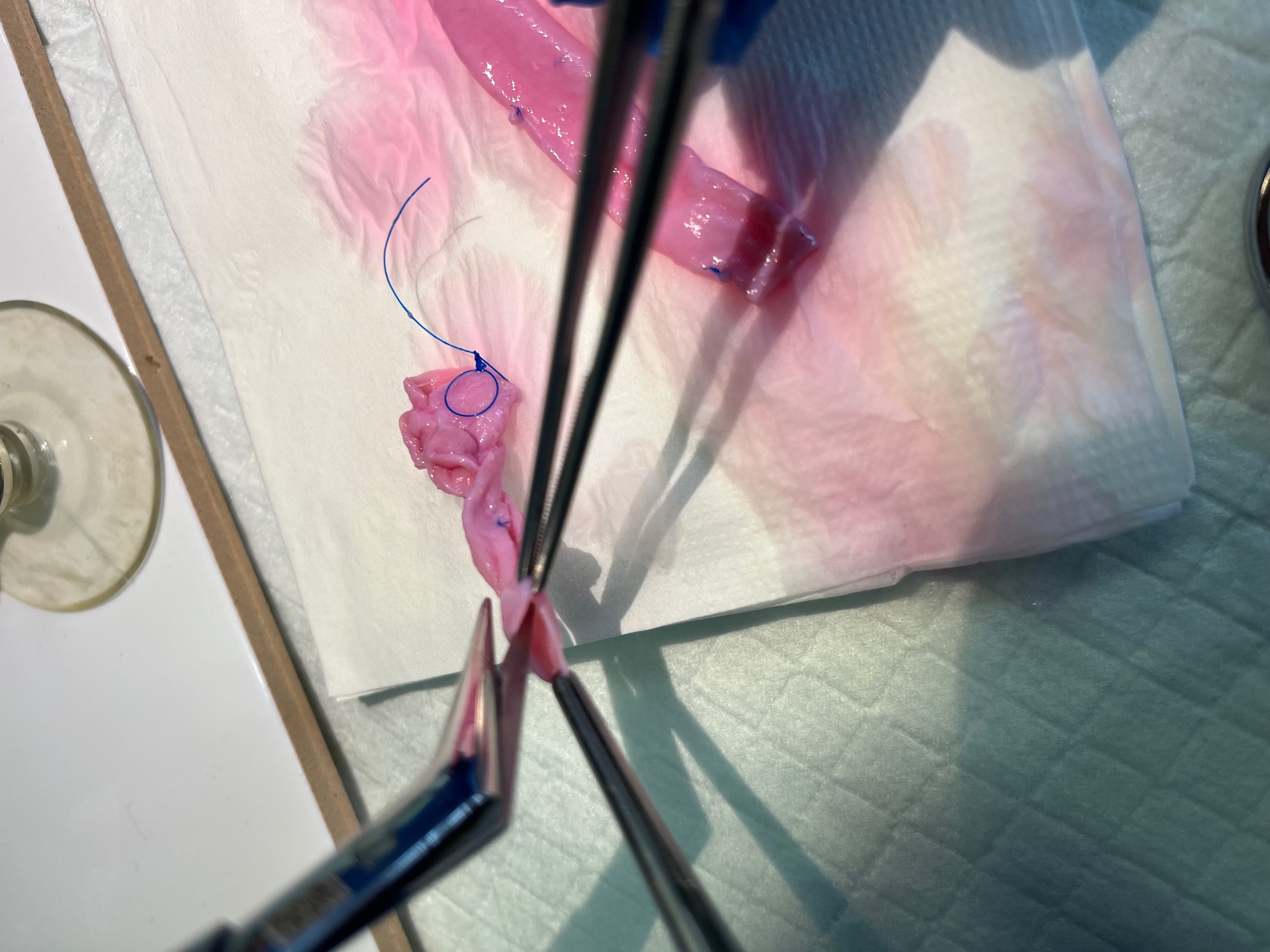


Figure 3B: Align the vein heels and toes


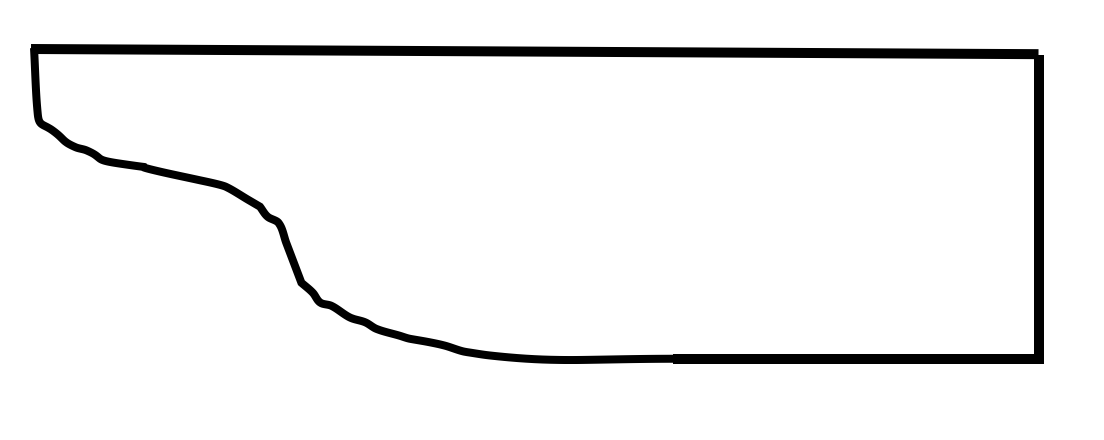

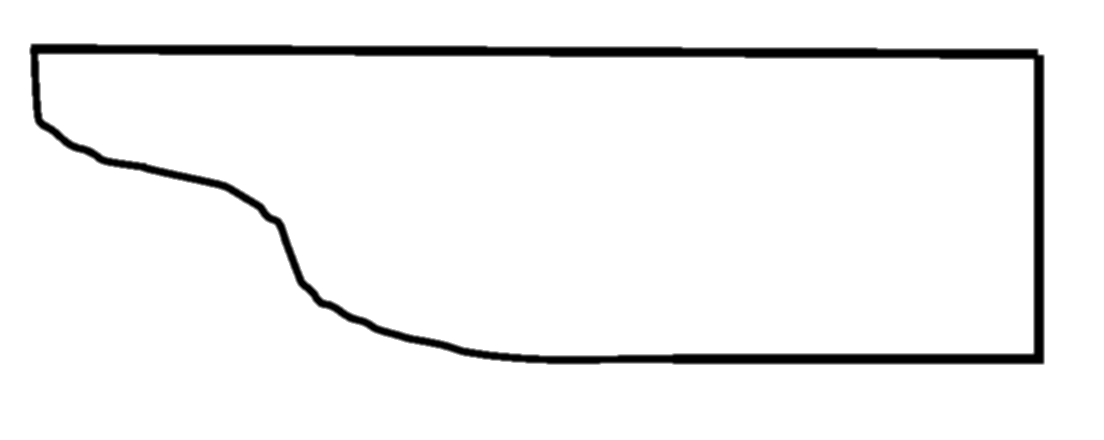


Picture 3C: Place stay sutures at the heel and toe and shod the ends

**
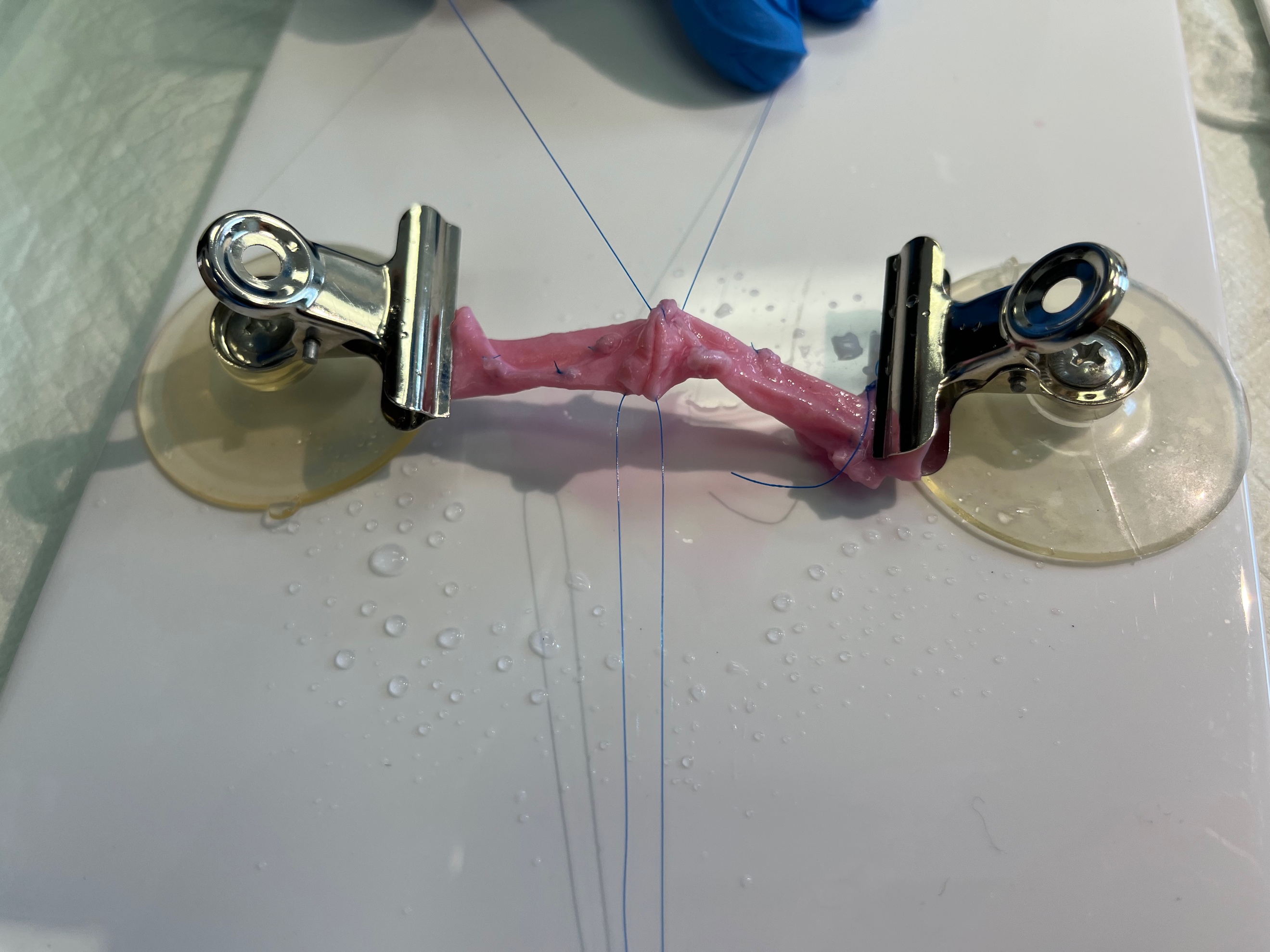
**

Picture 3D: Cannulate the vein and check for leaks with a red dye solution. Repair leaks with polypropylene stitches

**
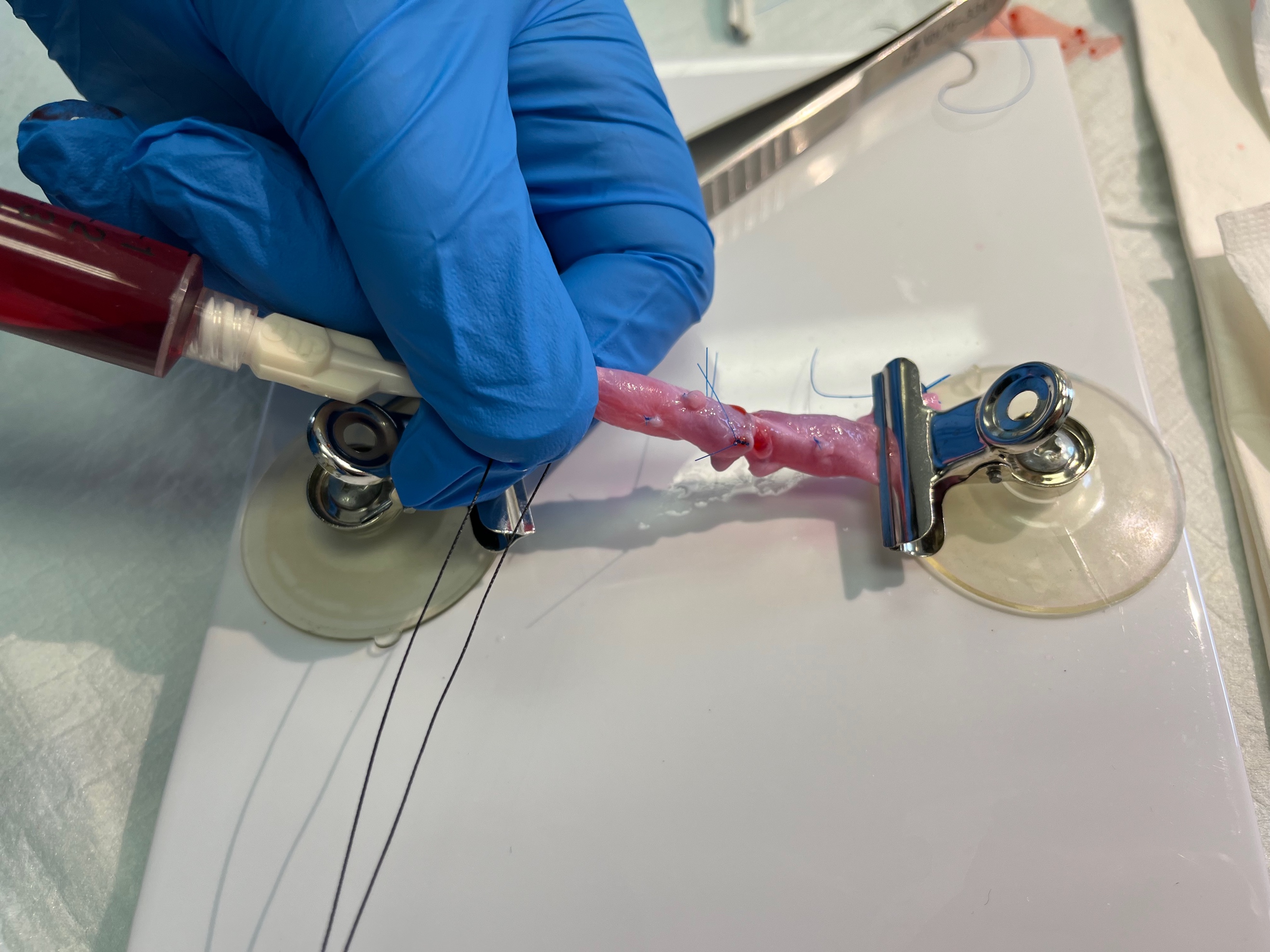
**
