# Supplementary material for: Vascular Anastomoses and Dissection: A Six-Part Simulation Curriculum for Surgical Residents
Source: MedEdPORTAL. 2024 May 28;20:11406. doi: 10.15766/mep_2374-8265.11406 (PMC11219091; doi:10.15766/mep_2374-8265.11406)
Supplement: Supplementary file 1 — Session 1 - End-to-End Anastomoses.docxSession 2 - End-to-Side Anastomoses.docxSession 3 - Cadaveric Vein Anastomoses.docxSession 4 - Aortic Exposure and Anastomosis.docxSession 5 - Vein Harvest.docxSession 6 - Extremity Bypass.docxSurveys.docx [file mep_2374-8265.11406-s001.zip › F. Session 6 - Extremity Bypass.docx]

**Appendix F: Session Six Details**

*Use this appendix to plan and execute the sixth session of the curriculum.*

*Pictures contained in this appendix are author owned.*

**Extremity Bypass**

***Summary:*** ***This two-hour session involves dissecting out the brachial artery in a porcine model and performing a short bypass using cadaveric vein. Residents will integrate skills from prior sessions, including dissection and vascular anastomoses with a tissue model.***

***Objectives:***

By the end of the session, residents should be able to:

- Identify and dissect out the distal brachial artery and proximal ulnar artery in a porcine model without creating holes or stenoses in the artery.
- Perform a proximal anastomosis to the brachial artery and distal anastomosis to the ulnar artery after vascular control is obtained.
- Assist the dissecting or sewing surgeon by providing retraction and exposure.

***Equipment:***

We use standard skills lab supplies (*), materials obtained through donation (^†^), and purchased materials (^‡^) for this session. The following should be available for each pair of trainees:

- Fine needle driver (e.g., Castro or Ryder/BM27)*
- Fine pickups (e.g., Gerald or fine DeBakey) x3*
- Rubber shod x4*
- Right angle*
- Tonsil clamps x2*
- Metzenbaum scissors*
- 10 and 11 blade scalpels*
- Porcine tissue model of a forelimb (Picture 6A)^‡^
- Cadaveric vein (4cm length)^†^
- 6-0 polypropylene (e.g., Prolene or Surgipro) suture x4*
- 4-0 silk ties*

***Set Up:***

- Before the session, email residents with session objectives, steps, and tips/tricks. Optionally, advise them to bring Loupes if available.
- Recruit vascular surgical faculty and/or advanced trainees (e.g., fellows) to circulate during the session and provide assistance.
- Place tissue model and remaining materials at each well-lit station.

***Session Steps and Timeline:***

- Introduce trainees to the objectives and task steps (5 minutes).
- Incise the medial surface of the forelimb using the 10 blade and dissect down to identify the brachial artery (Picture 6B) (10 minutes).
- Isolate the brachial artery (Picture 6C) and ulnar artery by dissecting away the perivascular tissue and controlling the branches. If vessel injury occurs, repair injuries with 6-0 polypropylene sutures (15 minutes).
- Spatulate the vein on both ends (Picture 6D) (5 minutes).
- Make an arteriotomy in the proximal and distal areas of exposed brachial and ulnar arteries using the 11 blade scalpel and scissors (5 minutes).
- Perform the proximal anastomosis (35 minutes).
  - Begin by placing a stitch at the heel (Picture 6E). Tie down with three throws. Place a stay suture at the toe to align the anastomosis for optimal needle angle and access, and shod the ends. Additional side stay stitches may be necessary for exposure.
  - Sew starting from the heel and proceeding along the circumference of the anastomosis. Adjust the vein with forceps to create the correct angles.
  - Repeat this technique for the second half of the anastomosis (Picture 6F). The two ends should meet at least two bites away from the toe.
- Time permitting, perform the distal anastomosis by repeating the above steps. A parachuting technique may be helpful given more limited exposure (i.e., do not initially tie down the heel stitch) (35 minutes).
- Perform a debrief with all residents to discuss challenges and lessons learned (10 minutes).

***Tips and Tricks:***

- Encourage tissue triangulation to expedite dissection.
- Stay in the proper plane by dissecting right along the brachial artery, taking care not to injury the vein or nerves.
- Tie in atraumatic fashion when ligating branches to prevent avulsion.
- Sutures can be placed around vessels as they are identified to assist with retraction.

Picture 6A: Porcine forelimb with area for incision indicated


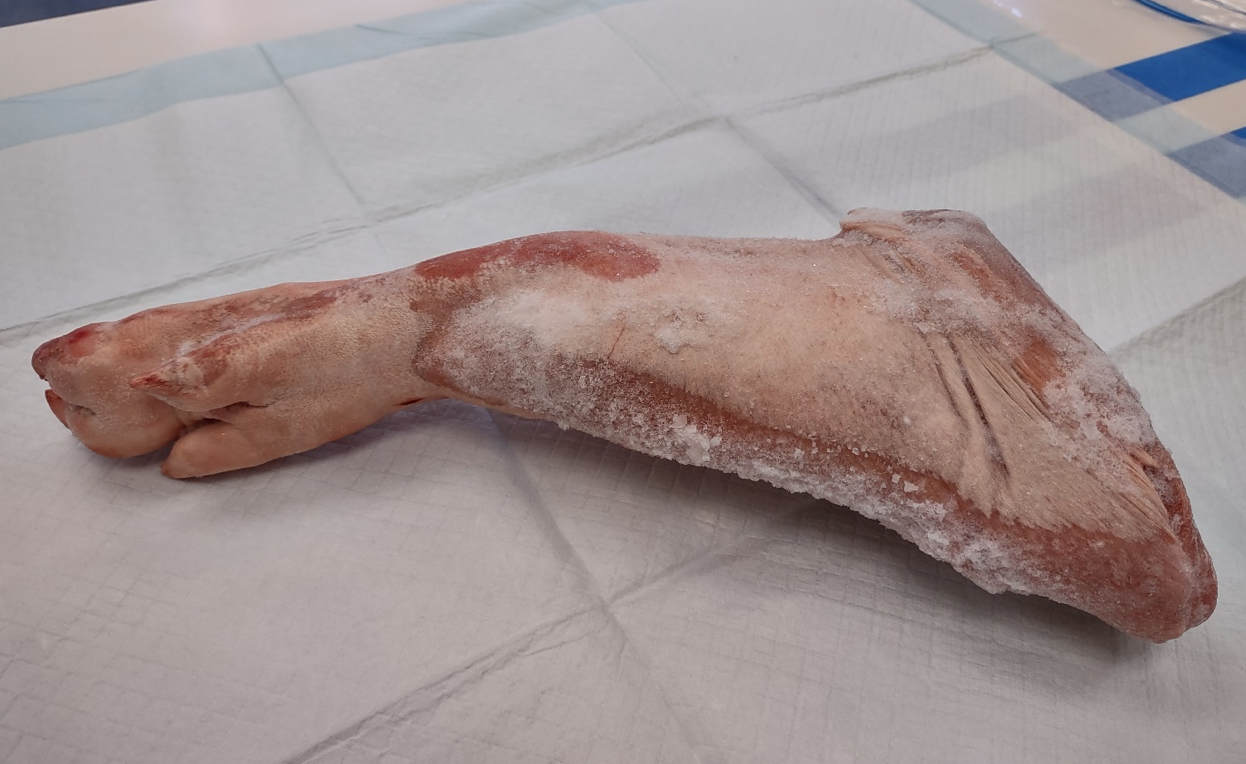


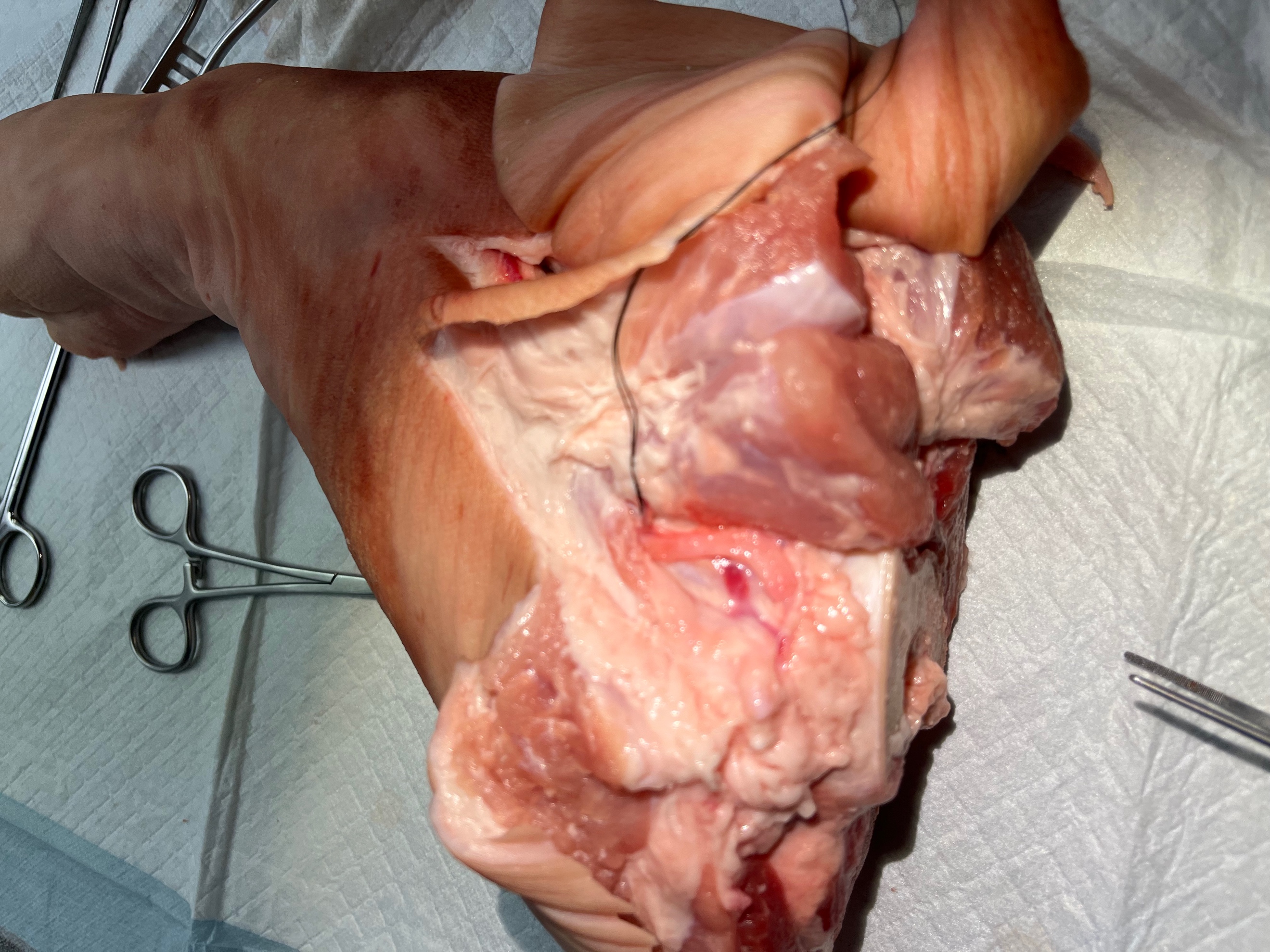


Picture 6B: Incision to expose brachial vessels

Picture 6C: Isolate the brachial artery (A) from the vein and nerve (N)


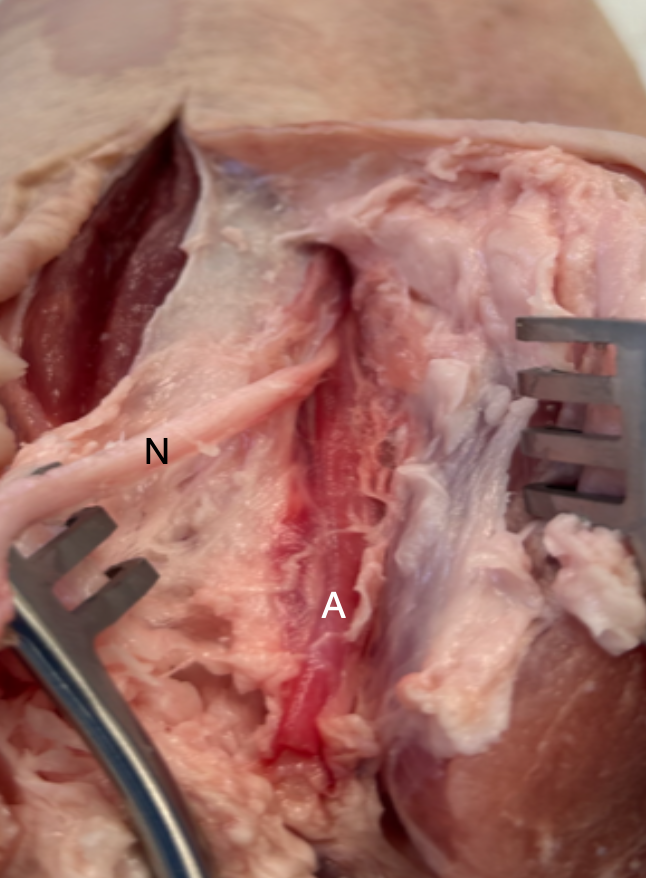


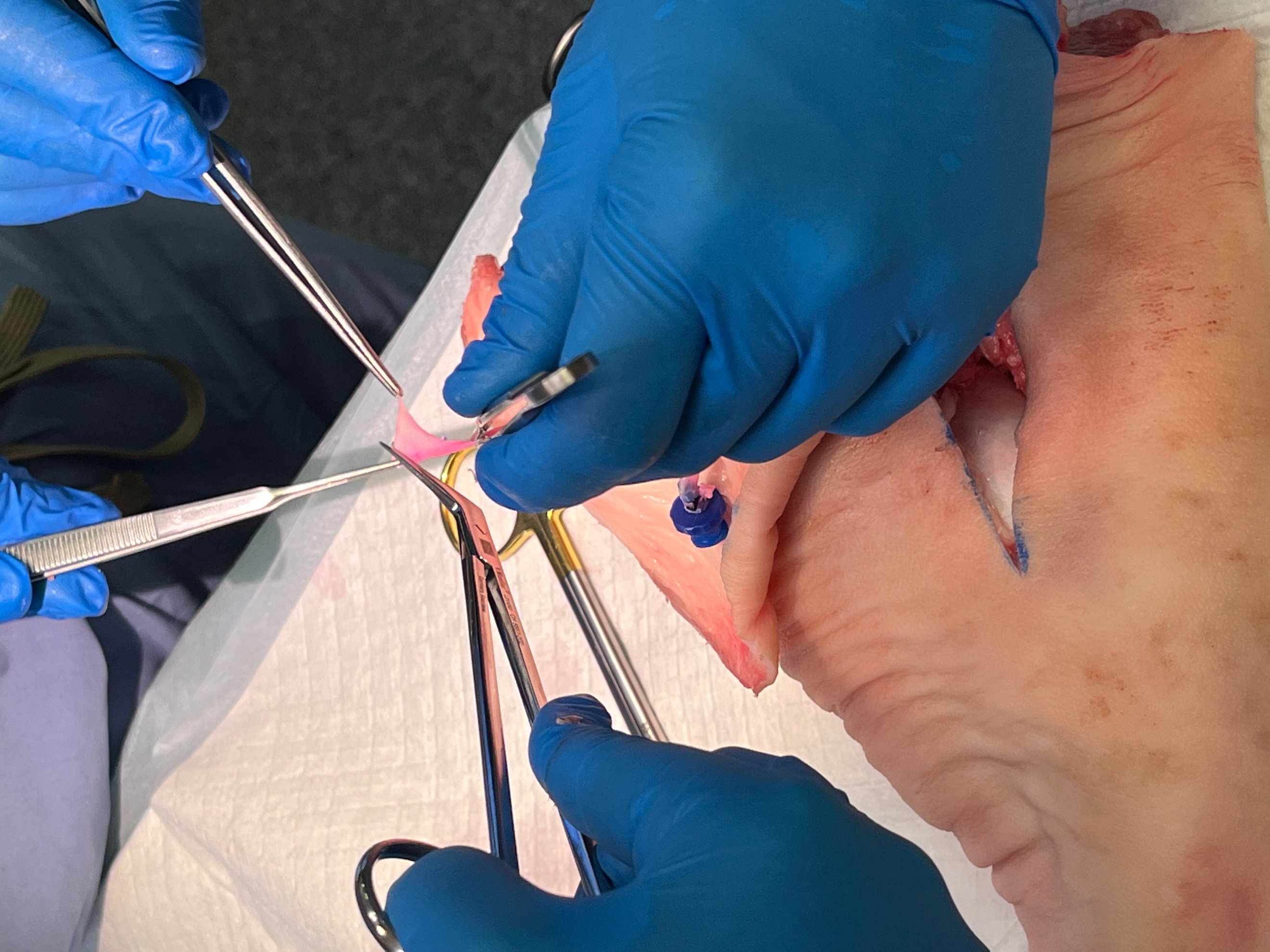


Picture 6D: Spatulate the ends of the vein

Picture 6E: Start the anastomosis with a stitch at the heel


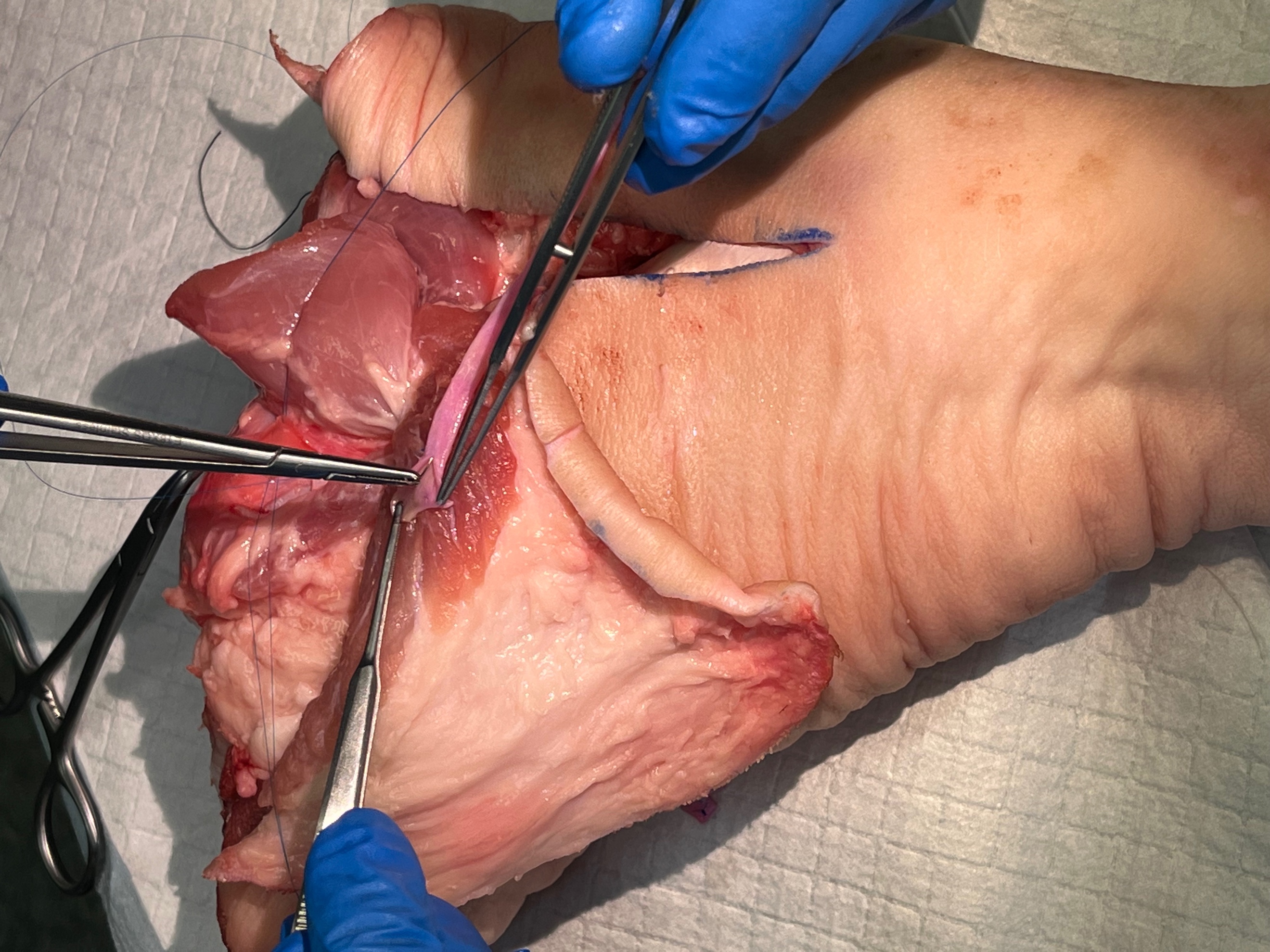


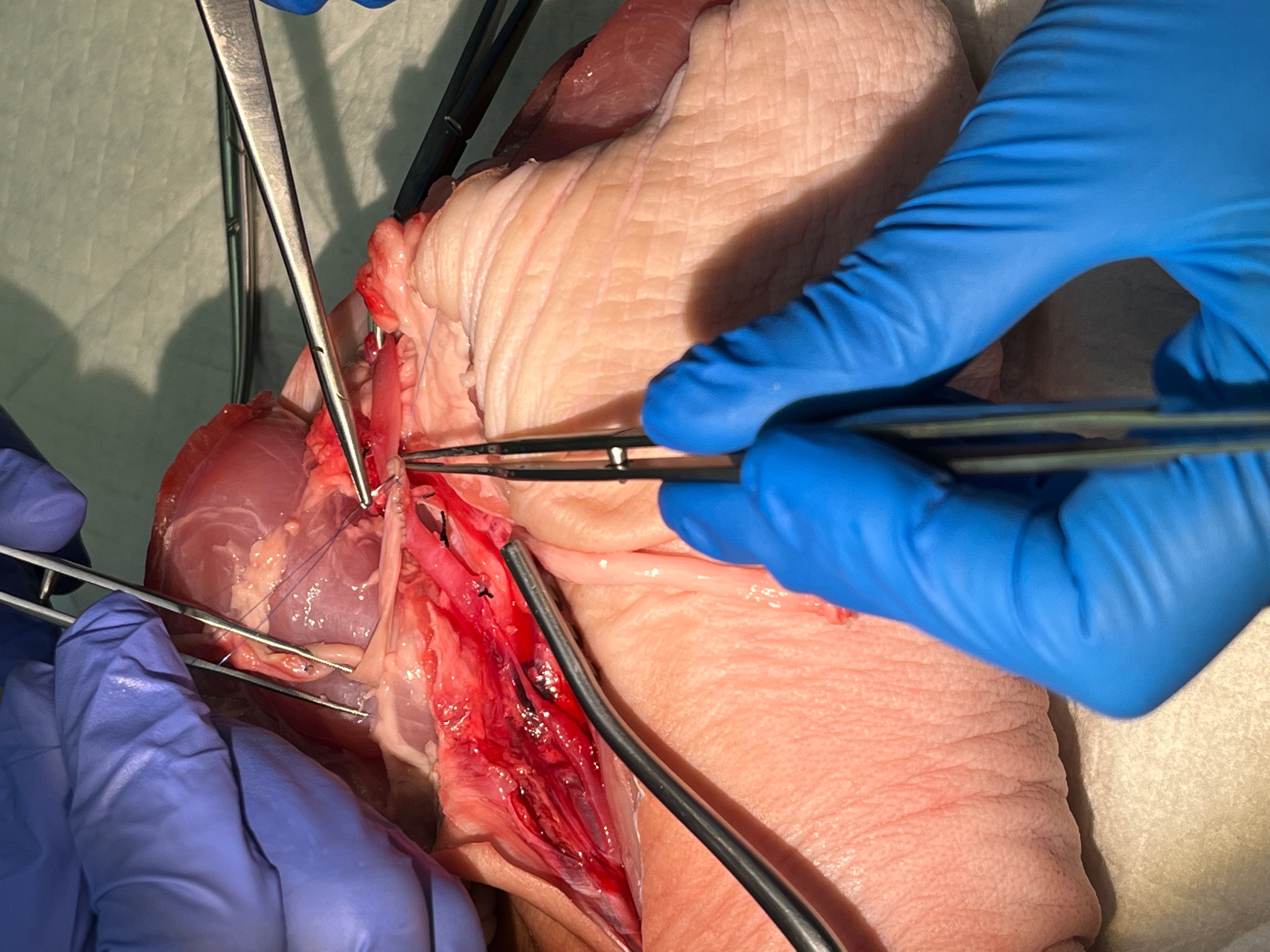


Picture 6F: Complete both sides of the anastomosis and tie away from the corner
