# Supplementary material for: Vascular Anastomoses and Dissection: A Six-Part Simulation Curriculum for Surgical Residents
Source: MedEdPORTAL. 2024 May 28;20:11406. doi: 10.15766/mep_2374-8265.11406 (PMC11219091; doi:10.15766/mep_2374-8265.11406)
Supplement: Supplementary file 1 — Session 1 - End-to-End Anastomoses.docxSession 2 - End-to-Side Anastomoses.docxSession 3 - Cadaveric Vein Anastomoses.docxSession 4 - Aortic Exposure and Anastomosis.docxSession 5 - Vein Harvest.docxSession 6 - Extremity Bypass.docxSurveys.docx [file mep_2374-8265.11406-s001.zip › G. Surveys.docx]

**Appendix G: Surveys**

*Use the first survey in this appendix to evaluate the curriculum. The second survey is provided for reference as it informed our earlier curricular iterations.*

**Vascular Curriculum-Specific Survey**

How would you rate the vascular skills lab experience?

- Poor
- Fair
- Good
- Very good
- Excellent

Please select your level of agreement with the following statements:

|  | Strongly disagree | Disagree | Neither agree nor disagree | Agree | Strongly agree |
| --- | --- | --- | --- | --- | --- |
| The 1:1 teaching in the vascular skills lab improved my ability to perform anastomoses with tissue |  |  |  |  |  |
| The 1:1 teaching in the vascular skills lab improved my ability to perform anastomoses with prosthetic |  |  |  |  |  |
| The 1:1 teaching in the vascular skills lab improved my ability to dissect |  |  |  |  |  |
| The 1:1 teaching in the vascular skills lab improved my vascular surgery skill set |  |  |  |  |  |
| The vascular surgery skills lab has taught me skills that will be useful throughout the rest of my general surgery residency |  |  |  |  |  |
| Skills lab models that are more “true to life” lead to better improvements in surgical skills |  |  |  |  |  |
| I am interested in vascular surgery as a career |  |  |  |  |  |

What was your favorite part(s) of the vascular skills labs?

|  |
| --- |

What are areas for improvement for the vascular skills labs?

|  |
| --- |

**General Curriculum Survey**

Please rate the following in terms of their educational quality for the Vascular Surgery sessions

|  | Poor | Below Average | Average | Above Average | Excellent |
| --- | --- | --- | --- | --- | --- |
| Didactics/ Presentations |  |  |  |  |  |
| Practical Exercises |  |  |  |  |  |
| Individual Instruction |  |  |  |  |  |

Please rate the following in terms of their educational quality for the Bowel Anastomosis sessions

|  | Poor | Below Average | Average | Above Average | Excellent |
| --- | --- | --- | --- | --- | --- |
| Didactics/ Presentations |  |  |  |  |  |
| Practical Exercises |  |  |  |  |  |
| Individual Instruction |  |  |  |  |  |

Please rate the following in terms of their educational quality for the Laparoscopy sessions

|  | Poor | Below Average | Average | Above Average | Excellent |
| --- | --- | --- | --- | --- | --- |
| Didactics/ Presentations |  |  |  |  |  |
| Practical Exercises |  |  |  |  |  |
| Individual Instruction |  |  |  |  |  |

My knowledge of surgical techniques has improved as a result of the skills lab

- Strongly disagree
- Disagree
- Neutral
- Agree
- Strongly agree

My technical abilities and technique have improved as a result of the skills lab

- Strongly disagree
- Disagree
- Neutral
- Agree
- Strongly agree

I use the knowledge and skills acquired in the lab in the OR, ER, clinic, or wards

- Strongly disagree
- Disagree
- Neutral
- Agree
- Strongly agree

I feel more comfortable with my skills knowledge and abilities as a result of the skills lab

- Strongly disagree
- Disagree
- Neutral
- Agree
- Strongly agree

The course moves at an appropriate pace for my abilities

- Strongly disagree
- Disagree
- Neutral
- Agree
- Strongly agree

I receive enough feedback to assess how I can improve

- Strongly disagree
- Disagree
- Neutral
- Agree
- Strongly agree

What could be done to help you learn/ improve from the R2 skills lab sessions?

|  |
| --- |

What two things do you like the best about the R2 skills lab/ R2 skills curriculum?

|  |
| --- |

What two things would you change about the R2 skills lab/ R2 skills curriculum?

|  |
| --- |

If we were to create an R3 skills curriculum, what should it include?

|  |
| --- |

Please write any additional comments, criticisms, or concerns.

|  |
| --- |
