# Supplementary material for: Vascular Anastomoses and Dissection: A Six-Part Simulation Curriculum for Surgical Residents
Source: MedEdPORTAL. 2024 May 28;20:11406. doi: 10.15766/mep_2374-8265.11406 (PMC11219091; doi:10.15766/mep_2374-8265.11406)

**Appendix A: Session One Details**

*Use this appendix to plan and execute the first session of the curriculum.*

*Pictures contained in this appendix are author owned.*

**End-to-End Anastomoses with PTFE**

***Summary:*** ***This two-hour session involves an end-to-end anastomosis with PTFE superficially and then at depth. Residents will work on basic vascular suturing skills by controlling needle angle, optimizing set up of the graft material, and assisting in retraction and “following.”***

***Objectives:***

By the end of the session, residents should be able to:

- Join two pieces of PTFE graft without twisting or narrowing the graft.
- Adjust the needle angle to be at a right angle to the anastomotic line throughout the entirety of the anastomosis.
- Space the needle “bites” to be even and consistent.
- Perform these activities superficially and at depth.

***Equipment:***

We use standard skills lab supplies (*) and materials obtained through donation (^†^) for this session. The following should be available for each pair of trainees:

- Fine needle driver (e.g., Castro or Ryder/BM27)*
- Fine pickups (e.g., Gerald or fine DeBakey) x3*
- Rubber shod x2*
- Metzenbaum scissors*
- Ceramic tile*
- Suction cup clips x2*
- PTFE (6-8cm length of 6-10mm graft)^†^
- Plastic box with central hole cut out*
- 5-0 or 6-0 polypropylene (e.g., Prolene or Surgipro) suture x4*

***Set Up:***

- Before the session, email residents with session objectives, steps, and tips/tricks. Optionally, advise them to bring Loupes if available.
- Recruit vascular surgical faculty and/or advanced trainees (e.g., fellows) to circulate during the session and provide assistance.
- Attach suction cup with clips opposite from each other on the ceramic tile.
- Place remaining materials at each well-lit station.

***Session Steps and Timeline:***

- Introduce trainees to the objectives and task steps (5 minutes).
- Cut the PTFE into two pieces with flat, straight edges. Clip each length of PTFE into a suction cup such that the ends meet in the middle (5 minutes).
- Begin the anastomosis by placing stay sutures at 0 and 180 degrees along the anastomotic line (Picture 1A). Use the stay sutures to align the anastomosis for optimal needle angle and access (Picture 1B) (10 minutes).
- Sew using one of the stay sutures to the midpoint between the stay sutures, change direction and use the other stay suture to complete the first half of the anastomosis (20 minutes).
  - Repeat this technique for the second half of the anastomosis to form the end product (Picture 1C) (20 minutes).
- Time permitting, repeat the above steps with the ceramic tile placed at the bottom of the box to simulate the anastomosis at depth (Picture 1D) (50 minutes).
- Perform a debrief and review the end product with all residents to discuss challenges and lessons learned (10 minutes).

***Tips and Tricks:***

- Change the position of the needle in the needle holder to create the ideal stitch angle.
- The assistant surgeon should keep tension on the suture and hold the edges of the graft to present the suturing surgeon the optimal exposure and needle angle.
- The assistant should follow with the “49-51” principle, in which the assistant manages almost half of the suture (49%) to give the surgeon enough length (51%) without the suture getting in the way.

Picture 1A: Place stay sutures at 0 and 180 degrees


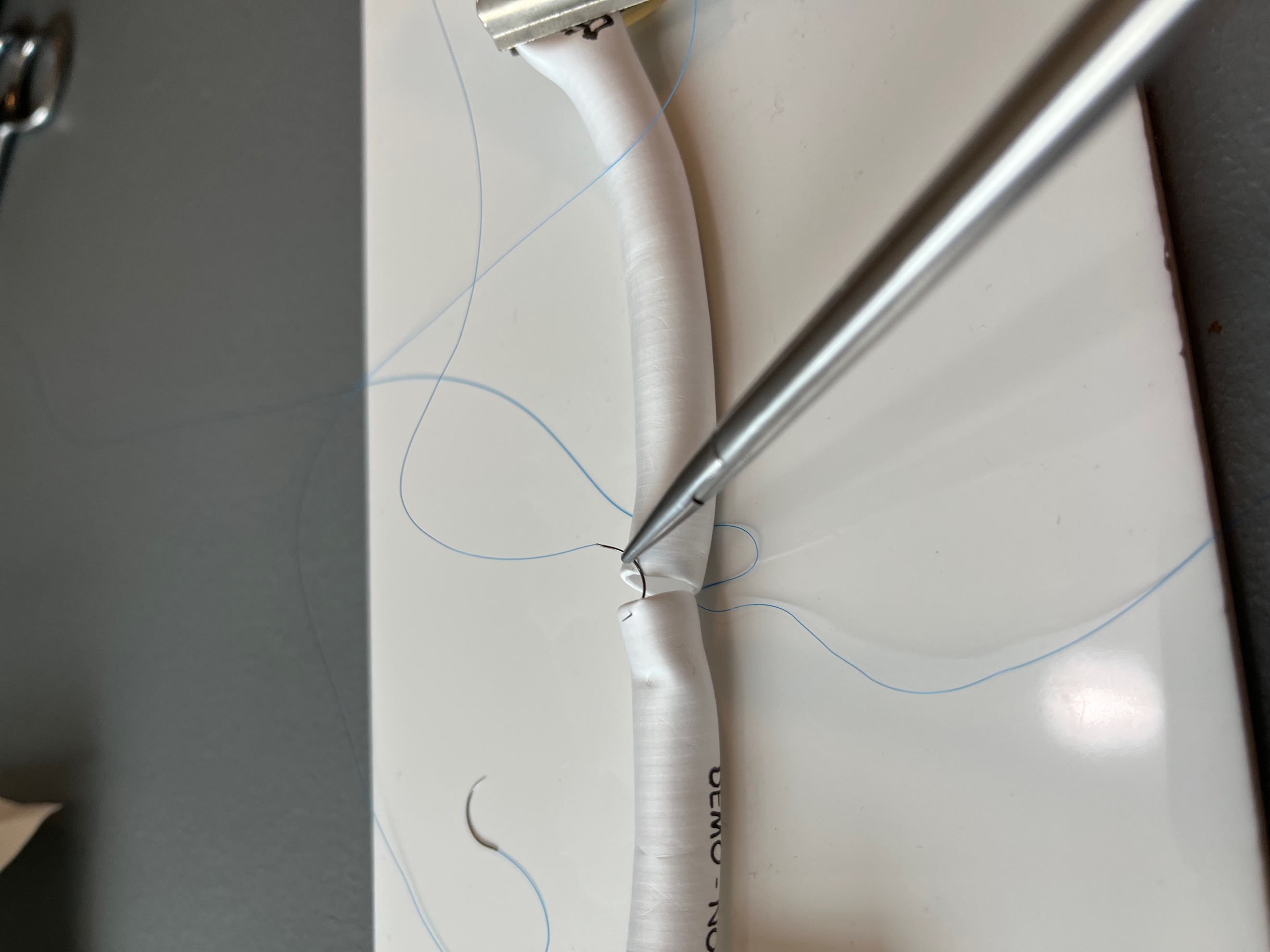


Picture 1B: Align the anastomosis


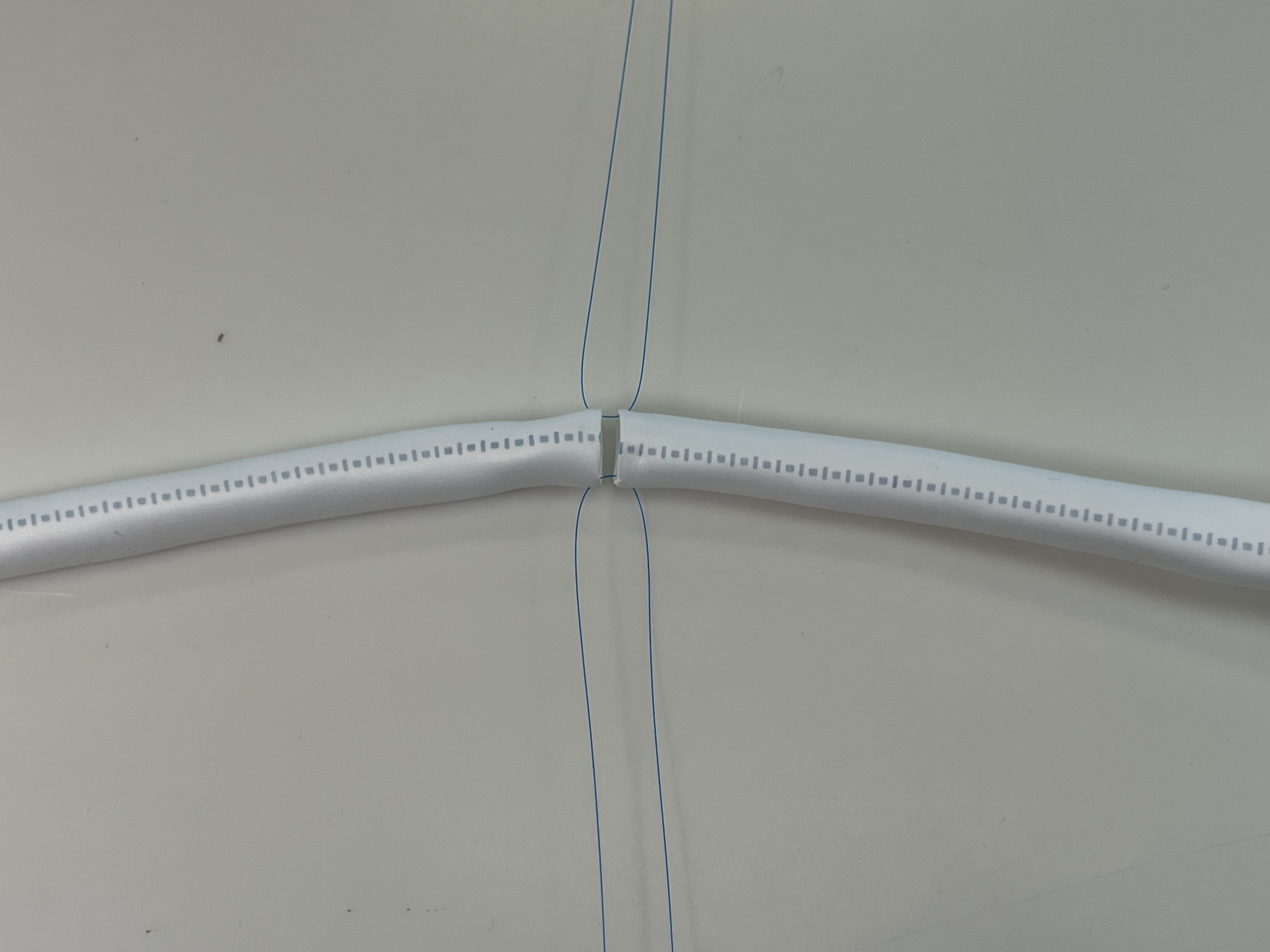


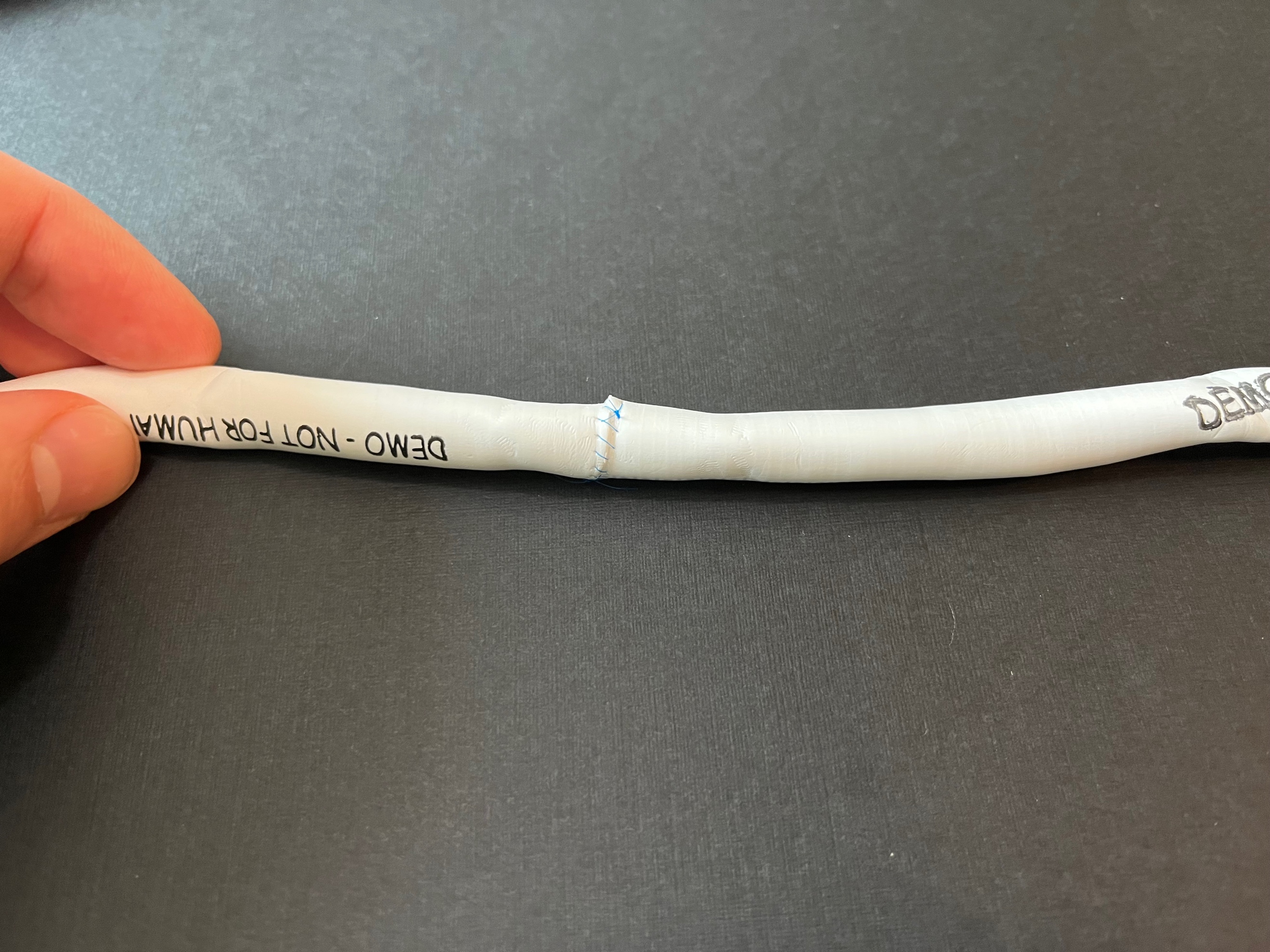


Picture 1C: End-to-end anastomosis

Picture 1D: Repeat the anastomosis at depth


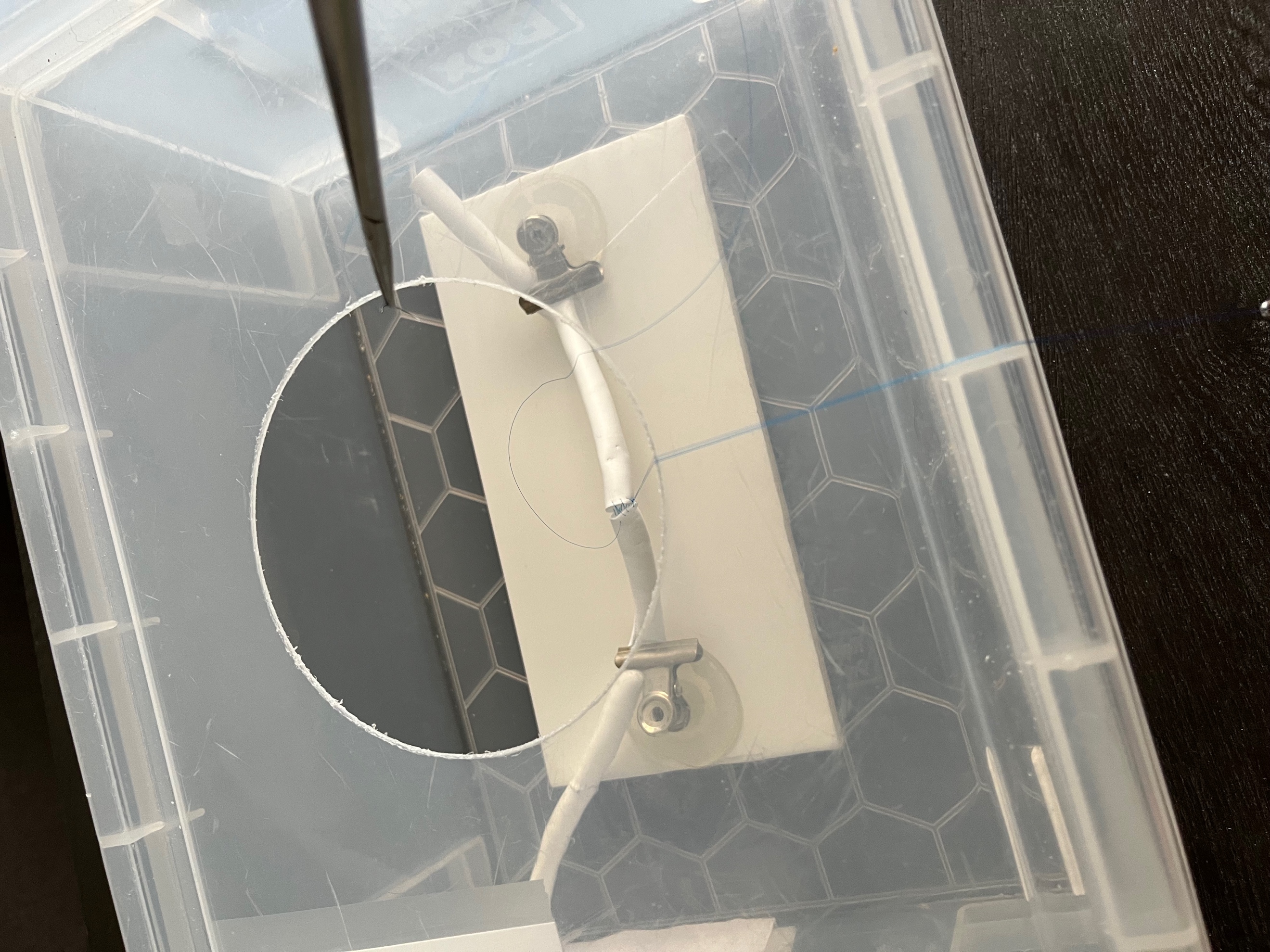

Supplement: Supplementary file 1 — Session 1 - End-to-End Anastomoses.docxSession 2 - End-to-Side Anastomoses.docxSession 3 - Cadaveric Vein Anastomoses.docxSession 4 - Aortic Exposure and Anastomosis.docxSession 5 - Vein Harvest.docxSession 6 - Extremity Bypass.docxSurveys.docx [file mep_2374-8265.11406-s001.zip › A. Session 1 - End-to-End Anastomoses.docx]
