# Supplementary material for: Vascular Anastomoses and Dissection: A Six-Part Simulation Curriculum for Surgical Residents
Source: MedEdPORTAL. 2024 May 28;20:11406. doi: 10.15766/mep_2374-8265.11406 (PMC11219091; doi:10.15766/mep_2374-8265.11406)

**Appendix B: Session Two Details**

*Use this appendix to plan and execute the second session of the curriculum.*

*Pictures contained in this appendix are author owned.*

**End-to-Side Anastomoses with PTFE and Parachute Technique**

***Summary:*** ***This two-hour session involves an end-to-side anastomosis with PTFE superficially and then at depth. Residents will work on basic vascular suturing skills by controlling needle angle, optimizing set up of the graft material, and assisting in retraction and “following.”***

***Objectives:***

By the end of the session, residents should be able to:

- Join two pieces of PTFE graft in an end-to-side fashion without twisting or narrowing the graft.
- Adjust the needle angle as indicated for the heel, toe, and sides of the anastomosis to be at a right angle to the anastomotic line.
- Space the needle “bites” to be even and consistent.
- Perform these activities superficially in the standard fashion and at depth using a parachute technique to facilitate initiating the anastomosis in a restricted space.

***Equipment:***

We use standard skills lab supplies (*) and materials obtained through donation (^†^) for this session. The following should be available for each pair of trainees:

- Fine needle driver (e.g., Castro or Ryder/BM27)*
- Fine pickups (e.g., Gerald or fine DeBakey) x3*
- Rubber shod x4*
- Metzenbaum scissors*
- 11 blade scalpel*
- Ceramic tile*
- Suction cup clips x2*
- PTFE (8cm length of 6-10mm graft, cut in half)^†^
- Plastic box with central hole cut out*
- 5-0 or 6-0 polypropylene (e.g., Prolene or Surgipro) suture x4*

***Set Up:***

- Before the session, email residents with session objectives, steps, and tips/tricks. Optionally, advise them to bring Loupes if available.
- Recruit vascular surgical faculty and/or advanced trainees (e.g., fellows) to circulate during the session and provide assistance.
- Attach suction cups to the ceramic tile and place one of the two pieces of PTFE between the clips.
- Place remaining materials at each well-lit station.

***Session Steps and Timeline:***

- Introduce trainees to the objectives and task steps (5 minutes).
- Make an “arteriotomy” in the side of the clipped piece of PTFE using the 11 blade scalpel and scissors. Cut the end of the second piece of PTFE into an Ogee curve to broaden the “heel and toe” (5 minutes).
- Begin the anastomosis by placing a stitch at the heel (Picture 2A). Tie down the first stitch with three throws and shod the ends. Place a stay suture at the toe to align the anastomosis for optimal needle angle and access (Picture 2B), and shod the ends (10 minutes).
- Sew starting from the heel and proceeding along the circumference of the anastomosis. Adjust the graft with forceps to create the correct angles. (20 minutes).
  - Repeat this technique for the second half of the anastomosis. The two ends should meet at least two bites away from the toe to form the end product (Picture 2C) (20 minutes).
- Time permitting, repeat the above steps with the ceramic tile placed at the bottom of the box to simulate the anastomosis at depth (Picture 2D). Use the parachute technique, in which the heel and toe stitches are kept long and not initially tied (50 minutes).
- Perform a debrief and review the end product with all residents to discuss challenges and lessons learned. If residents performed both the first tie-down technique and the parachute technique, contrast experiences with the two methods (10 minutes).

***Tips and Tricks:***

- Change the position of the needle in the needle holder to create the ideal stitch angle.
- The assistant should keep tension on the suture and hold the edges of the graft to present the operator the optimal exposure and needle angle.
- The assistant should follow with the “49-51” principle as previously described and take care to manage the suture length when parachuting to avoid tangling.
- Review the importance of going inside to outside on the artery (as opposed to the vein) in the operating room to prevent plaque disruption.

Picture 2A: Start the anastomosis at the heel


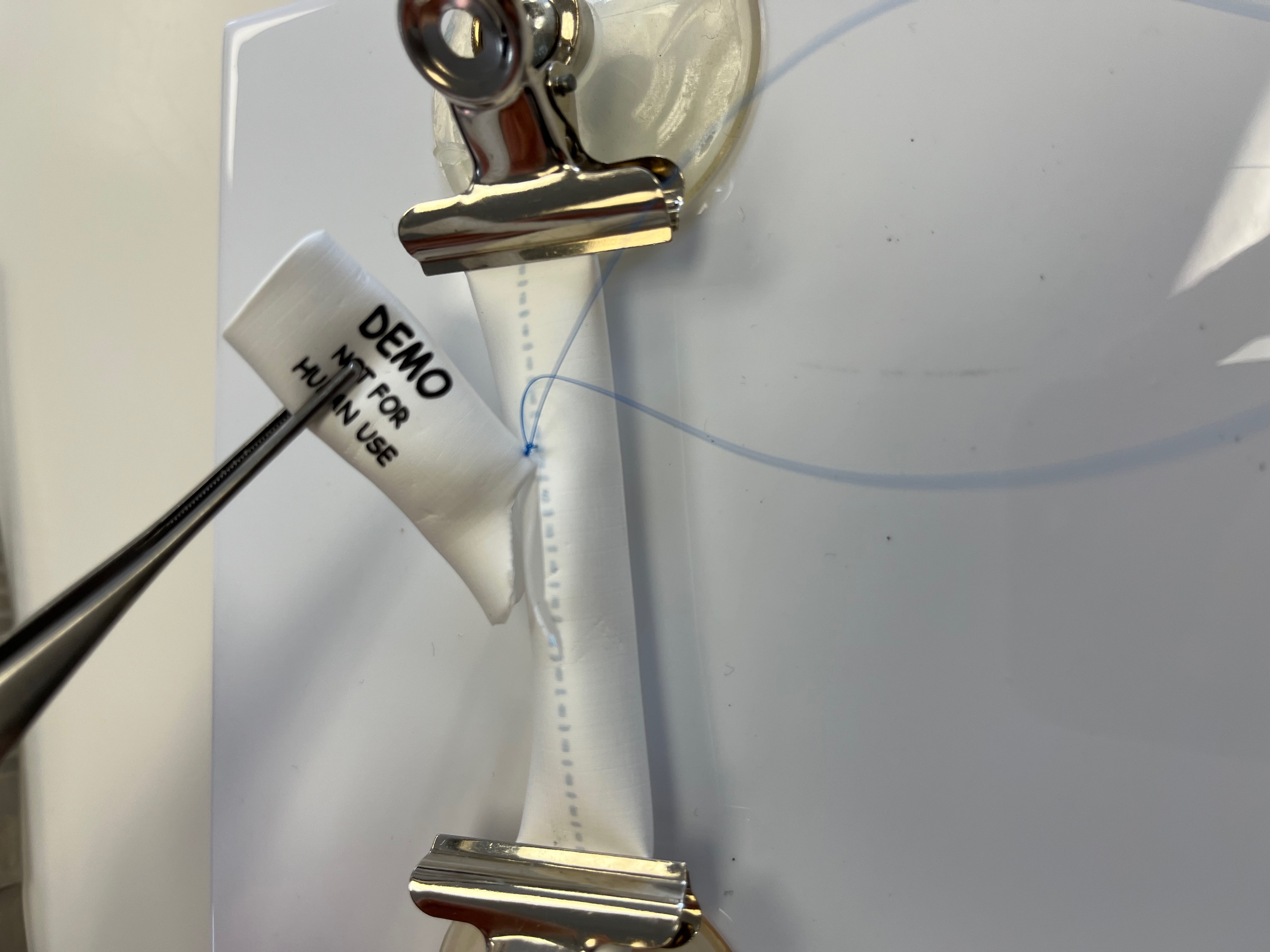


Picture 2B: Place a stay stitch at the toe


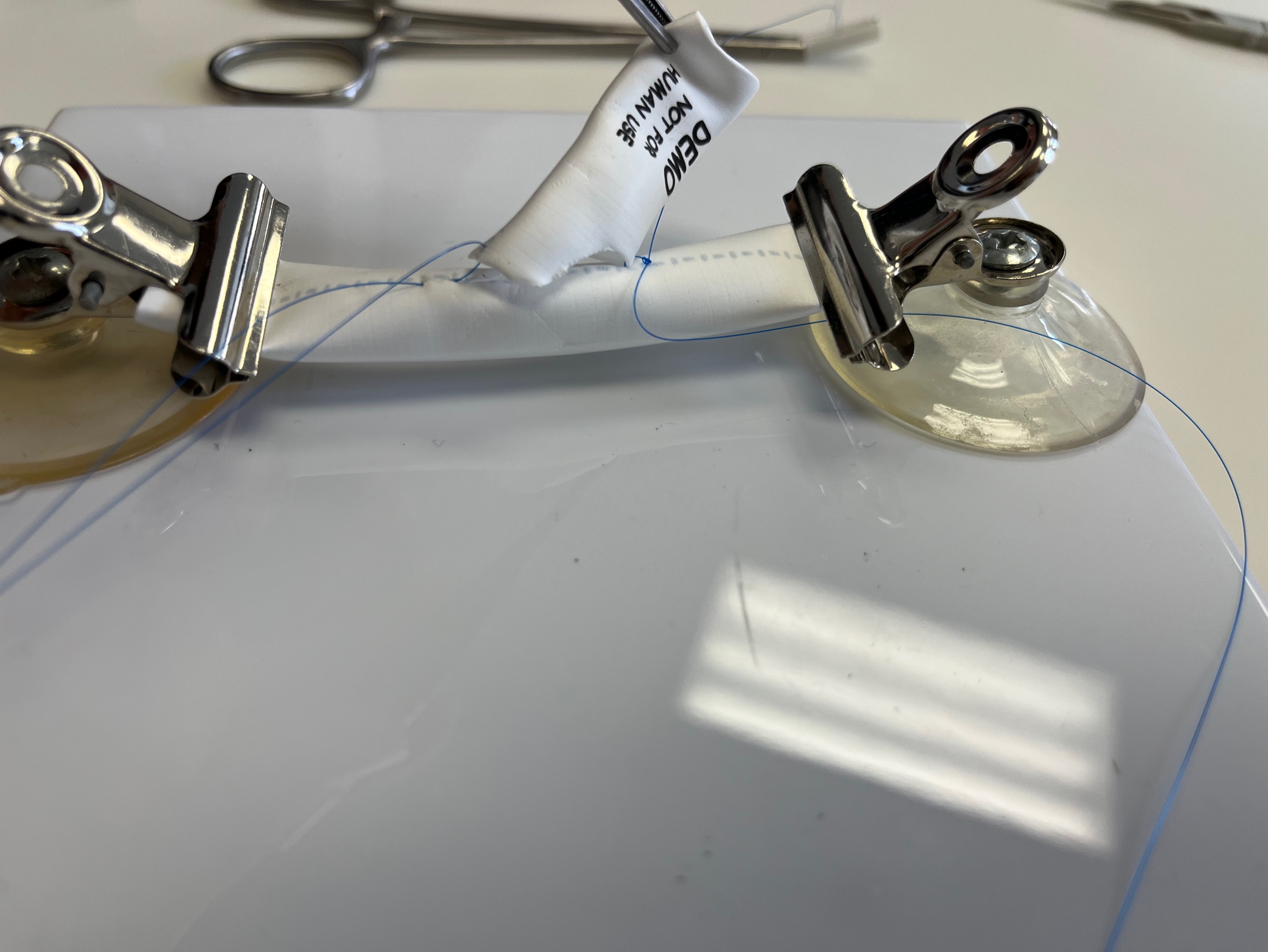


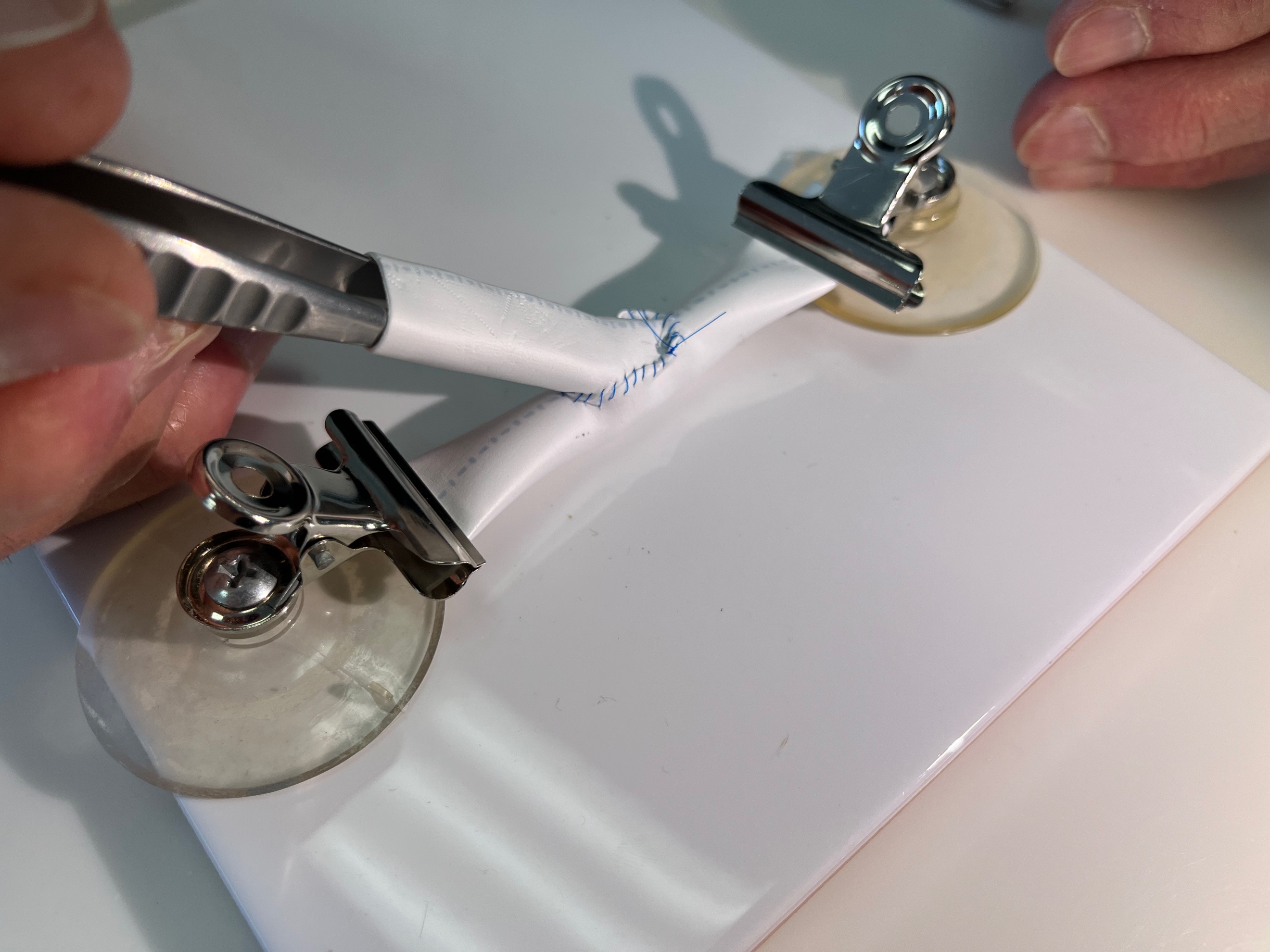


Picture 2C: End-to-side anastomosis

Picture 2D: Repeat the exercise at depth using the parachuting technique


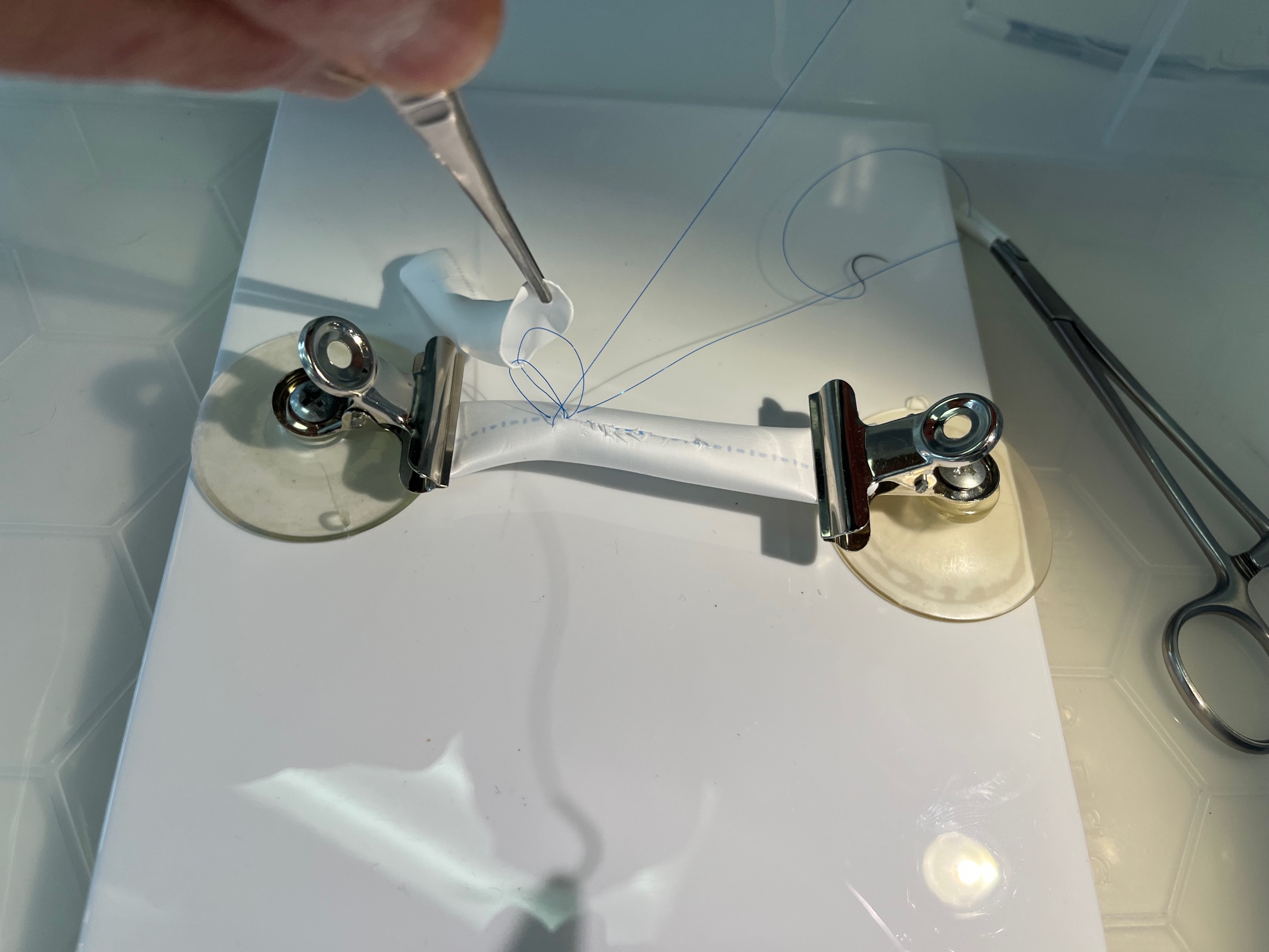

Supplement: Supplementary file 1 — Session 1 - End-to-End Anastomoses.docxSession 2 - End-to-Side Anastomoses.docxSession 3 - Cadaveric Vein Anastomoses.docxSession 4 - Aortic Exposure and Anastomosis.docxSession 5 - Vein Harvest.docxSession 6 - Extremity Bypass.docxSurveys.docx [file mep_2374-8265.11406-s001.zip › B. Session 2 - End-to-Side Anastomoses.docx]
