# Supplementary material for: Vascular Anastomoses and Dissection: A Six-Part Simulation Curriculum for Surgical Residents
Source: MedEdPORTAL. 2024 May 28;20:11406. doi: 10.15766/mep_2374-8265.11406 (PMC11219091; doi:10.15766/mep_2374-8265.11406)
Supplement: Supplementary file 1 — Session 1 - End-to-End Anastomoses.docxSession 2 - End-to-Side Anastomoses.docxSession 3 - Cadaveric Vein Anastomoses.docxSession 4 - Aortic Exposure and Anastomosis.docxSession 5 - Vein Harvest.docxSession 6 - Extremity Bypass.docxSurveys.docx [file mep_2374-8265.11406-s001.zip › _Educational Summary Report_11406.pdf]

MedEdPORTAL® The AAMC Journal of Teaching and Learning Resources

Original Publication Open Access

Vascular Anastomoses and Dissection: A Six-Part Simulation Curriculum for Surgical Residents

Riley Brian, MD, MAEd*, Natalie Rodriguez, MD, Joseph Rapp, MD, Hueylan Chern, MD, Patricia O'Sullivan, EdD, Clara Gomez-Sanchez, MD

*Corresponding author: riley.brian@ucsf.edu

Abstract

Introduction: As surgical technologies grow, so too do demands on surgical trainees to master increasing numbers of skill sets. With the rise of endovascular surgery, trainees have fewer opportunities to practice open vascular techniques in the operating room. Simulation can bridge this gap. However, existing published open vascular simulation curricula are basic or based on expensive models. **Methods:** We iteratively developed an open vascular skills curriculum for second-year surgery residents comprising six 2-hour sessions. We refined the curriculum based on feedback from learners and faculty. The curriculum required skilled facilitators, vascular instruments, and tissue models. We evaluated the latest iteration with a survey and by assessing participants' technical skills using the Objective Structured Assessment of Technical Skills (OSATS) form. **Results:** Over the past 10 years, 101 residents have participated in the curriculum. Nine of 13 residents who participated in the latest curricular iteration completed the survey. All respondents rated the sessions as excellent and strongly agreed that they had improved their abilities to perform anastomoses with tissue and prosthetic. Facilitators completed 18 OSATS forms for residents in the fifth and sixth sessions of the latest iteration. Residents scored well overall, with a median 26.5 (interquartile range: 24-29) out of a possible score of 35, with highest scores on knowledge of instruments. **Discussion:** This simulation-based curriculum facilitates open vascular surgical skill acquisition among surgery residents. The curriculum allows residents to acquire critical vascular skills that are challenging to learn in an increasingly demanding operative setting.

Keywords
Open Vascular Skills, Surgical Simulation, Deliberate Practice, Cardiovascular Medicine, Clinical/Procedural Skills Training, Simulation, Surgery - Vascular

Educational Objectives

By the end of this activity, learners will be able to:

1. Perform vascular anastomoses as the operating surgeon for superficial and deep simulated procedures.
2. Assist in providing retraction and exposure as the assisting surgeon for simulated vascular anastomoses.
3. Dissect both arteries and veins from perivascular tissue.

Introduction

Surgical training poses increasing demands for residents to gain experience in broad techniques, with mastery expected in open, laparoscopic, robotic, endoscopic, and endovascular techniques.[1] In vascular surgery, endovascular techniques have supplanted open approaches in many areas.[2,3] Similarly, the rise of integrated vascular surgery residency programs may affect vascular surgical training for general surgery trainees, particularly as such programs grow in prevalence and maturity.[4] A 2016 study found that operative volumes for all major types of open vascular surgery declined between 54% and 83% from 1999 to 2012, and this trend has likely continued.[5] Despite the decline in operative opportunity, the Accreditation Council for Graduate Medical Education expects general surgery residents to become proficient in certain open vascular techniques.[6]

As operative experience changes, simulation takes a central role in training for vital but less commonly performed procedures. Simulation is an effective method by which to provide open surgical training, and prior authors have credited simulation as "an indispensable tool" in vascular surgery.[7,8] A nationwide study in the United States showed that though a significant majority of trainees found value in vascular simulation, almost half lacked access to formal simulation.[9] Surgical simulation is based on the

Citation:
Brian R, Rodriguez N, Rapp J, Chern H, O'Sullivan P, Gomez-Sanchez C. Vascular anastomoses and dissection: a six-part simulation curriculum for surgical residents. *MedEdPORTAL*. 2024;20:11406.
https://doi.org/10.15766/mep_2374-8265.11406

Copyright © 2024 Brian et al. This is an open-access publication distributed under the terms of the Creative Commons Attribution license.

theory of deliberate practice, which posits that carefully designed training with feedback, problem solving, and opportunities for repeated performance allows for the acquisition of expert skills.[10] Deliberate practice is widely cited in the medical and surgical education literatures as foundational to physician and surgeon growth.[11,12] Timing of the simulation can benefit from an understanding of the zone of proximal development, in which instruction is most useful when the task being performed is just beyond the learner's current capabilities.[13] This allows for appropriate task selection and timing in surgical training.

Simulation models exist for open vascular surgery. Many models are prohibitively expensive; for example, a vascular simulation model described by Pandey and Wolfe in 2012 costs $2,600.[14,15] While there are more affordable models, many are resource intensive in other ways or have limited their scope to a single procedure or part of a procedure.[16-18] One curriculum for basic open vascular training has been published in *MedEdPORTAL,* though this focuses on instrument knowledge and foundational skills.[19] Other published curricula center on endovascular surgery, abdominal aortic aneurysm repair, or carotid endarterectomy.[20] Vascular anastomosis curricula have been evaluated, though prior models are complex to set up and publications lack the details necessary for implementation.[15,21] Few curricula incorporate vascular dissection.[20] As such, there is a gap in published curricula to facilitate the implementation of vascular anastomoses and dissection.

Methods

Development

At our institution, first- and second-year surgery residents participated in a longitudinal 2-year curriculum through our Surgical Skills Center (SSC) to develop surgical skills. Sessions were 2 hours every 2 weeks and were facilitated by surgical faculty. These sessions varied significantly, from basic knot tying and suturing skills for beginning participants to sophisticated laparoscopic skills for more advanced participants. Second-year residents participated in a vascular curriculum.

The roots of this vascular curriculum began over a decade ago. In 2013, for example, the second-year surgery residents completed a 10-session vascular curriculum with one to two sessions per month led by one vascular surgery faculty member. Since that time, we have held six to 10 sessions per year. At the end of each session, a debriefing was conducted with the participating residents to identify challenges and opportunities for improvement. After each session, the SSC team members debriefed the session. Team members included the session organizers, faculty present at the session, and residents assisting with the session. These meetings involved discussion of how to change and optimize each session in future iterations. The majority of vascular surgery faculty at our institution have participated in these sessions and have contributed perceptions and ideas to improve the curriculum's content and organization.

As a result of this iterative process, we refined six sessions focusing on skill acquisition by centering learning in the zone of proximal development (Figure 1). The six sessions covered end-to-end anastomoses with polytetrafluoroethylene (PTFE), end-to-side anastomoses with PTFE and parachute, cadaveric vein anastomoses, aortic exposure and anastomoses, vein harvest, and extremity bypass.

In sessions 1 and 2 (Appendices A and B), residents performed vascular anastomoses in pairs using synthetic grafts both superficially and at depth. These sessions equipped residents with skills such as needle angling, atraumatic tying, optimizing retraction, and working at depth. In sessions 3 and 4 (Appendices C and D), residents transitioned to using animal and cadaveric tissue to build on skills with real tissue and to learn basics of tissue handling and dissection. In sessions 5 and 6 (Appendices E and F), residents continued to expand on their tissue handling and began to learn advanced exposures and small vessel isolation.

Equipment/Environment

The sessions worked best in a well-lit environment with sufficient table space for the number of learners. Most materials for the sessions were standard instruments and equipment available in most surgical skills settings, such as fine pickups, clamps, and sutures. This curriculum could not have been feasibly implemented without such instruments and equipment. Porcine tissue was affordably obtained from a butcher or slaughterhouse (about $30 per learner for the whole curriculum). Cadaveric vein was obtained through a cryopreservation company (e.g., CryoLife). Specific material needs are listed for each session in Appendices A-F.

Personnel

Key personnel to perform this curriculum included an SSC operations manager or staff member with experience preparing surgical simulation sessions and one or more vascular surgery faculty members.

The SSC operations manager or staff member obtained the materials for each session and performed each task outlined in the setup sections of Appendices A-F. The vascular surgery

Copyright © 2024 Brian et al. This is an open-access publication distributed under the terms of the Creative Commons Attribution license.

Session Title	Needle Angling	Atraumatic Tying	Optimizing Retraction	Working at Depth	Tissue Handling/ Dissection	Advanced Exposure	Small Vessel Isolation
End-to-end anastomoses with PTFE	x	x	x	x			
End-to-side anastomoses with PTFE and parachute	x	x	x	x			
Cadaveric vein anastomoses	x	x	x	x	x		
Aortic exposure and anastomosis	x	x	x		x	x	
Vein harvest	x	x	x		x	x	x
Extremity bypass	x	x	x	x	x	x	x

Figure 1. Curricular sessions with associated skills. The curriculum aimed to promote graduated skill development by facilitating practice in the zone of proximal development. Abbreviation: PTFE, polytetrafluoroethylene.

faculty member(s) attended each session, introduced the key concepts to residents at the start of the session, circulated among residents during the session to provide immediate feedback, debriefed with the residents at the end of the session, and debriefed with other involved organizers after the session.

Implementation

The details of implementation for each of the six sessions are outlined in Appendices A-F. Each session took approximately 2 hours to complete.

Generally, setup started with the SSC operations manager or staff member obtaining the required materials for the session and confirming session time/date with the vascular surgery faculty. The operations manager or staff member also notified residents about the session time/date and provided them with the session objectives, procedural steps, and tips/tricks from the appropriate sections of Appendices A-F.

On the morning of a session, the SSC operations manager or staff member laid out the necessary materials per pair of residents. Tissue models were kept moist with a spray bottle. When the faculty and residents had arrived, the faculty introduced the session by briefly outlining the session steps, skills required, and tips for success. The residents then broke into pairs to perform the steps as outlined in the session steps and timeline sections of Appendices A-F. Paired residents alternated between sewing/dissecting and retracting/exposing. During this time, the faculty circulated among the residents and provided immediate feedback.

Debriefing

At the start of each session, the faculty communicated that the group would reconvene in the last 10 minutes of the session to discuss challenges and lessons learned. During these last 10 minutes, each participant described something they had found challenging about the session and a key takeaway that they would use in the next session and in the operating room. We also used this time for general comments on the session to improve its future implementation. Debriefing in a group setting allowed residents to understand their common challenges and hear how their colleagues approached these challenges.

Assessment

We performed learner assessment using the Objective Structured Assessment of Technical Skills (OSATS). Developed in 1997, the OSATS has been used extensively in surgical simulation.[22,23] It involved assigning a score from 1 to 5 in seven skill areas. Although other simulation-based assessment tools existed, we used the OSATS given its history in the assessment of open vascular skills and its ease of use.[24,25] We assessed with the OSATS during the fifth and sixth sessions.[22,23]

We performed curriculum evaluation and iterative improvement through debriefs with residents (Debriefing section, above) and

Copyright © 2024 Brian et al. This is an open-access publication distributed under the terms of the Creative Commons Attribution license.

the SSC team (Development section, above). Additionally, we administered surveys about our surgical skills curriculum as a whole at three points since 2016. These surveys included questions specific to the vascular curriculum regarding educational quality and areas for improvement. Responses allowed for iterative change each year. Finally, we administered a survey to residents following our latest iteration of the curriculum in 2022-2023 (Appendix G). We adapted this survey from a published questionnaire assessing residents' experience with a vascular simulation session.[15] We made adaptations to the survey to improve flow based on published survey development guidelines and discussed these adaptations with content experts.[26,27] Our Institutional Review Board exempted this curricular evaluation (UCSF IRB 22-37166, 2022).

Results

Since 2013, 101 second-year residents have participated in the vascular curriculum. Thirteen residents, that is, every second-year resident at our institution, participated in the most recent version of the curriculum from 2022 to 2023, as detailed here. These included nine general surgery residents, one integrated vascular surgery resident, and three plastic surgery residents. Seven vascular and transplant surgery faculty members and two general surgery research residents participated during at least one of the six sessions. All sessions had two or more faculty members and at least one research resident as facilitators. We reviewed session objectives, setup, and plans with lead facilitators in curriculum planning meetings prior to each session. During sessions, all resident participants successfully performed the anastomoses and dissections making up the curriculum.

We performed learner assessment using the OSATS. From the curriculum's latest iteration, facilitators completed 18 OSATS forms in the fifth and sixth sessions, during which all resident participants successfully harvested a vein and performed a bypass anastomosis. The median overall assigned OSATS score was 26.5 (interquartile range: 24-29) out of a possible score of 35 (Figure 2). Among the OSATS components, participants received the highest scores in knowledge of instruments and the lowest scores in time and motion (Table).

We conducted iterative curriculum evaluation using learner debriefing, general curriculum surveys (2017, 2021, and 2022), and a vascular curriculum–specific survey (2023). We modified the curriculum multiple times based on learner debriefing and comments from the general curriculum surveys. Most of the feedback was generically positive (e.g., "I enjoyed having multiple vascular sessions," "The vascular sessions are particularly helpful," and "Vascular labs were great"). However,

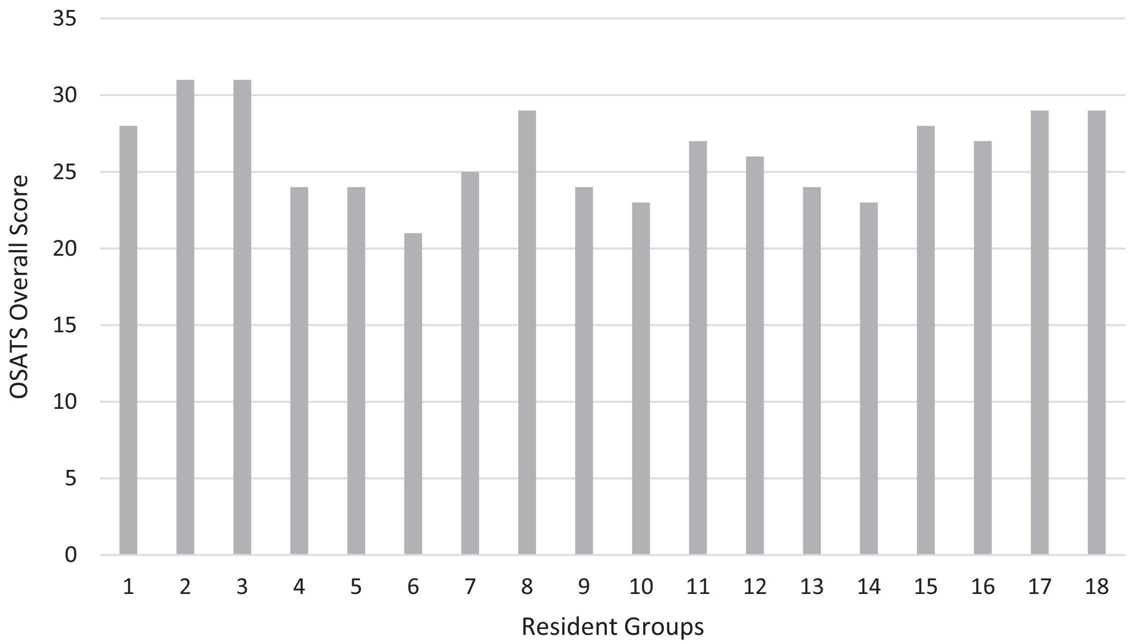

Figure 2. Overall scores on the Objective Structured Assessment of Technical Skills (OSATS) form. Facilitators rated resident groups on performance using the OSATS form, with an overall score out of 35. Eighteen forms were completed for sessions 5 and 6 during the curriculum's latest iteration (2022-2023).

Copyright © 2024 Brian et al. This is an open-access publication distributed under the terms of the Creative Commons Attribution license.

Table. Overall and Component Scores on the OSATS Form

OSATS Area[a]	*Mdn* (IQR)
Respect for tissue	4 (3-4)
Time and motion	3 (3-4)
Instrument handling	4 (3-4)
Knowledge of instruments	4 (4-5)
Use of assistants	4 (3-4)
Flow of operation and forward planning	3.5 (3-4)
Knowledge of specific procedure	4 (3-4)
Overall score	26.5 (24-29)

Abbreviations: IQR, interquartile range; OSATS, Objective Structured Assessment of Technical Skills.
[a]Facilitators rated residents on performance using the OSATS form. Each component was scored from 1 to 5 with an overall score out of 35. Facilitators completed a total of 18 forms for sessions 5 and 6 during the curriculum's latest iteration (2022-2023).

some respondents also provided specific comments about the curriculum. Much of this specific feedback was positive; for instance, one participant stated, "The introduction of new skills is always exciting and makes for an easier and less stressful transition to using those skills in the OR." Other feedback was more constructive. One 2017 survey participant asked for reference material to prepare for and reflect upon the vascular curriculum sessions. A 2021 survey respondent noted that having multiple faculty members present to provide feedback was helpful. We incorporated these suggestions and recommendations from debriefings into the latest version of the curriculum.

In the 2022-2023 iteration, nine of 13 participants (69%) completed the curriculum survey. All respondents rated the curriculum as excellent (Figure 3). All respondents strongly agreed that the vascular sessions taught them skills that would be useful throughout residency and that the one-on-one teaching in the vascular sessions improved their skill sets, their abilities to perform anastomoses with tissue, and their abilities to perform anastomoses with prosthetic. Three of these nine residents reported being interested in a career in vascular surgery. In constructed response items, residents described their favorite parts of the curriculum and areas for improvement. Residents were most appreciative of the one-on-one feedback, ability to repeat skills through the curriculum, and live fresh-tissue models. They suggested continuing to improve the curriculum by creating video demonstrations for the tasks and providing additional diagrams.

Discussion

Residents' open vascular surgical experience has declined even as proficiency in such open skills remains expected and required for safe patient care. Simulation for open vascular surgery has shown significant promise in bridging the gap between operative experience and expected skill.[7,8,20] However, we found no published curricula specifically detailing the several key skills required for open vascular surgical anastomosis and dissection in a simulation setting. Here, we have described such a curriculum.

Our curriculum is built on the theory of deliberate practice through its use of explicit session objectives, frequent individualized feedback, and thoughtful skill repetition.[11,12] The curriculum's learner selection builds on the idea of the zone of proximal development by enlisting second-year residents during the period in which they are beginning to build the necessary skills, including vascular sewing and dissection, for the tasks.[13] Each session aims to push residents' abilities slightly beyond

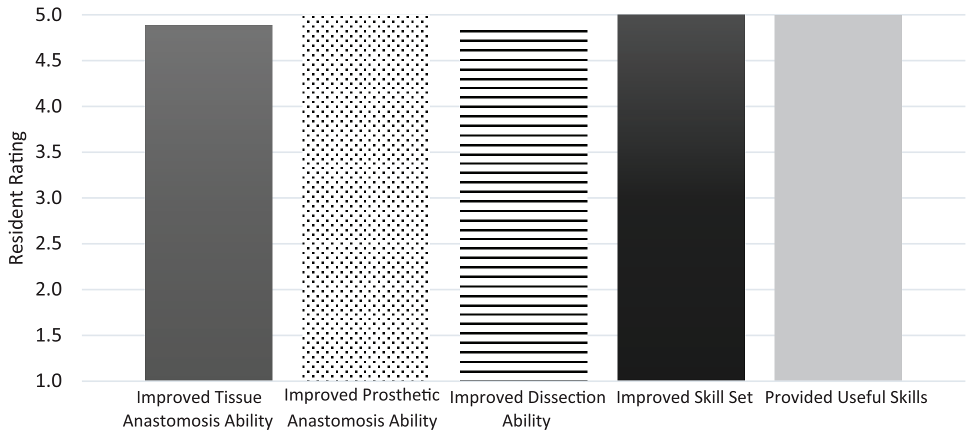

Figure 3. Resident perceptions of the latest iteration of the vascular curriculum. Residents who completed the entirety of the curriculum (sessions 1-6) in its latest iteration (2022-2023) assessed its components on a 5-point Likert scale (1 = *strongly disagree*, 5 = *strongly agree*) in several areas. Nine of 13 residents (69%) completed the survey.

Copyright © 2024 Brian et al. This is an open-access publication distributed under the terms of the Creative Commons Attribution license.

prior sessions, thereby striking an appropriate balance between known and unknown. Residents commented on the repeated hands-on experience with feedback as being helpful for their learning.

The version of the vascular simulation curriculum presented here has resulted from years of iterative development and interval changes. Over the past 10 years, feedback—both formal and informal—from residents and faculty has contributed to our curriculum. Lessons from prior years emphasized the importance of specific feedback, the usefulness of tissue models, and the utility of facilitator training. In its latest form, the curriculum was exceptionally well received by residents, who universally found the sessions to improve their skills and abilities in key areas. Assessment of residents' skills by facilitators using the OSATS form showed an overall high level of skill by the fifth and sixth simulation sessions. Together, these data suggest an effective curriculum. Residents did note additional areas for future improvement related to curricular video and visual materials. We plan to continue to improve the curriculum based on this feedback.

There are several key limitations to this curriculum. First, we lack data regarding actual operative and patient impact. We instead used the OSATS, which measures a number of the skills required to meet our Educational Objectives. While we expect many of these key skills to transfer to the operative setting, it was not feasible to perform intraoperative resident assessment following sessions given the high variability in rotation schedule and, thus, the opportunity to practice these skills on a predictable timeline. Furthermore, we lack the OSATS data from earlier iterations of the curriculum, which would have informed the curriculum's maturation over time. Additionally, the curriculum was implemented at a large academic medical center with multiple available experts; extra training for general surgery facilitators may be needed in settings without available experts. Finally, the curriculum requires several materials that may not be available in all locations. Most materials (e.g., instruments and sutures) are widely available in academic settings, though cadaveric vein required industry donation and tissue models came from butchers. The use of standard skills lab supplies and industry donation allowed us to implement this curriculum for only the cost of porcine tissue from butchers; if industry-donated materials are unavailable, the curriculum will need to be adjusted or shortened.

In conclusion, we have described a six-session curriculum facilitating open vascular surgical skill acquisition among second-year surgery residents. This simulation-based curriculum allows residents to acquire critical vascular skills that are challenging to learn in an increasingly demanding operative setting.

Appendices

A. Session 1 - End-to-End Anastomoses.docx
B. Session 2 - End-to-Side Anastomoses.docx
C. Session 3 - Cadaveric Vein Anastomoses.docx
D. Session 4 - Aortic Exposure and Anastomosis.docx
E. Session 5 - Vein Harvest.docx
F. Session 6 - Extremity Bypass.docx
G. Surveys.docx

All appendices are peer reviewed as integral parts of the Original Publication.

Riley Brian, MD, MAEd: Research Resident, Department of Surgery, University of California, San Francisco; ORCID: https://orcid.org/0000-0002-7462-3214

Natalie Rodriguez, MD: Third-Year Resident, Department of Surgery, University of California, San Francisco

Joseph Rapp, MD: Professor Emeritus of Surgery, Department of Surgery, University of California, San Francisco

Hueylan Chern, MD: Professor of Surgery, Department of Surgery, University of California, San Francisco

Patricia O'Sullivan, EdD: Professor of Medicine and Surgery, School of Medicine, University of California, San Francisco

Clara Gomez-Sanchez, MD: Assistant Professor of Surgery, Department of Surgery, University of California, San Francisco

Disclosures
None to report.

Funding/Support
None to report.

Ethical Approval
The University of California, San Francisco, Institutional Review Board deemed further review of this project not necessary.

References

1. Toale C, O'Byrne A, Morris M, Kavanagh DO. Defining operative experience targets in surgical training: a systematic review. *Surgery*. 2022;172(5):1364-1372. https://doi.org/10.1016/j.surg.2022.07.015
2. Suckow BD, Goodney PP, Columbo JA, et al. National trends in open surgical, endovascular, and branched-fenestrated

Copyright © 2024 Brian et al. This is an open-access publication distributed under the terms of the Creative Commons Attribution license.

endovascular aortic aneurysm repair in Medicare patients. *J Vasc Surg*. 2018;67(6):1690-1697.E1. https://doi.org/10.1016/j.jvs.2017.09.046

3. Dansey KD, Varkevisser RRB, Swerdlow NJ, et al. Epidemiology of endovascular and open repair for abdominal aortic aneurysms in the United States from 2004 to 2015 and implications for screening. *J Vasc Surg*. 2021;74(2):414-424. https://doi.org/10.1016/j.jvs.2021.01.044
4. Carroll MI, Downes K, Miladinovic B, et al. A single-institution experience: the integrated vascular surgery residency's effect on fellowship and general surgery resident case volume and diversity. *Ann Vasc Surg*. 2014;28(1):253-259. https://doi.org/10.1016/j.avsg.2013.03.010
5. Krafcik BM, Sachs TE, Farber A, et al. Assessment of open operative vascular surgical experience among general surgery residents. *J Vasc Surg*. 2016;63(4):1110-1115. https://doi.org/10.1016/j.jvs.2015.09.062
6. Review Committee for Surgery. *Defined Category Minimum Numbers for General Surgery Residents and Credit Role.* Accreditation Council for Graduate Medical Education; 2019. Accessed April 19, 2024. https://www.acgme.org/globalassets/definedcategoryminimumnumbersforgeneralsurgeryresidentsandcreditrole.pdf
7. Davies J, Khatib M, Bello F. Open surgical simulation—a review. *J Surg Educ*. 2013;70(5):618-627. https://doi.org/10.1016/j.jsurg.2013.04.007
8. Bismuth J, Donovan MA, O'Malley MK, et al. Incorporating simulation in vascular surgery education. *J Vasc Surg*. 2010;52(4):1072-1080. https://doi.org/10.1016/j.jvs.2010.05.093
9. Duran C, Bismuth J, Mitchell E. A nationwide survey of vascular surgery trainees reveals trends in operative experience, confidence, and attitudes about simulation. *J Vasc Surg*. 2013;58(2):524-528. https://doi.org/10.1016/j.jvs.2012.12.072
10. Krampe RT, Ericsson KA. Maintaining excellence: deliberate practice and elite performance in young and older pianists. *J Exp Psychol Gen*. 1996;125(4):331-359. https://doi.org/10.1037/0096-3445.125.4.331
11. Anders Ericsson K. Deliberate practice and acquisition of expert performance: a general overview. *Acad Emerg Med*. 2008; 15(11):988-994. https://doi.org/10.1111/j.1553-2712.2008.00227.x
12. Higgins M, Madan CR, Patel R. Deliberate practice in simulation-based surgical skills training: a scoping review. *J Surg Educ*. 2021;78(4):1328-1339. https://doi.org/10.1016/j.jsurg.2020.11.008
13. Vygotsky LS. *Mind in Society: The Development of Higher Psychological Processes*. Harvard University Press; 1978.
14. Pandey VA, Wolfe JHN. Expanding the use of simulation in open vascular surgical training. *J Vasc Surg*. 2012;56(3):847-852. https://doi.org/10.1016/j.jvs.2012.04.015
15. Bartline PB, O'Shea J, McGreevy JM, Mueller MT. A novel perfused porcine simulator for teaching aortic anastomosis increases resident interest in vascular surgery. *J Vasc Surg*. 2017;66(2):642-648.E4. https://doi.org/10.1016/j.jvs.2017.01.049
16. Moorthy K, Munz Y, Adams S, Pandey V, Darzi A; Imperial College—St. Mary's Hospital Simulation Group. Self-assessment of performance among surgical trainees during simulated procedures in a simulated operating theater. *Am J Surg*. 2006;192(1):114-118. https://doi.org/10.1016/j.amjsurg.2005.09.017
17. Robinson WP III, Schanzer A, Cutler BS, et al. A randomized comparison of a 3-week and 6-week vascular surgery simulation course on junior surgical residents' performance of an end-to-side anastomosis. *J Vasc Surg*. 2012;56(6):1771-1781. https://doi.org/10.1016/j.jvs.2012.06.105
18. Robinson WP, Doucet DR, Simons JP, et al. An intensive vascular surgical skills and simulation course for vascular trainees improves procedural knowledge and self-rated procedural competence. *J Vasc Surg*. 2017;65(3):907-915.E3. https://doi.org/10.1016/j.jvs.2016.12.065
19. Morcos O, Schindler N, Singer M. Introduction to vascular instruments and techniques skills lab curriculum. *MedEdPORTAL*. 2014;10:9655. https://doi.org/10.15766/mep_2374-8265.9655
20. Haiser A, Aydin A, Kunduzi B, Ahmed K, Dasgupta P. A systematic review of simulation-based training in vascular surgery. *J Surg Res*. 2022;279:409-419. https://doi.org/10.1016/j.jss.2022.05.009
21. Wilasrusmee C, Phromsopha N, Lertsitichai P, Kittur DS. A new vascular anastomosis model: relation between outcome and experience. *Eur J Vasc Endovasc Surg*. 2007;33(2):208-213. https://doi.org/10.1016/j.ejvs.2006.09.026
22. Martin JA, Regehr G, Reznick R, et al. Objective structured assessment of technical skill (OSATS) for surgical residents. *Br J Surg*. 1997;84(2):273-278. https://doi.org/10.1046/j.1365-2168.1997.02502.x
23. Hatala R, Cook DA, Brydges R, Hawkins R. Constructing a validity argument for the Objective Structured Assessment of Technical Skills (OSATS): a systematic review of validity evidence. *Adv Health Sci Educ Theory Pract*. 2015;20(5):1149-1175. https://doi.org/10.1007/s10459-015-9593-1
24. Glarner CE, McDonald RJ, Smith AB, et al. Utilizing a novel tool for the comprehensive assessment of resident operative performance. *J Surg Educ*. 2013;70(6):813-820. https://doi.org/10.1016/j.jsurg.2013.07.009
25. Larson JL, Williams RG, Ketchum J, Boehler ML, Dunnington GL. Feasibility, reliability and validity of an operative performance rating system for evaluating surgery residents. *Surgery*. 2005;138(4):640-649. https://doi.org/10.1016/j.surg.2005.07.017

Copyright © 2024 Brian et al. This is an open-access publication distributed under the terms of the Creative Commons Attribution license.

26. Sullivan GM, Artino AR Jr. How to create a bad survey instrument. *J Grad Med Educ*. 2017;9(4):411-415. https://doi.org/10.4300/JGME-D-17-00375.1

27. Artino AR Jr, La Rochelle JS, Dezee KJ, Gehlbach H. Developing questionnaires for educational research: AMEE Guide no. 87. *Med Teach*. 2014;36(6):463-474. https://doi.org/10.3109/0142159X.2014.889814

Received: June 14, 2023
Accepted: February 28, 2024
Published: May 28, 2024

Copyright © 2024 Brian et al. This is an open-access publication distributed under the terms of the Creative Commons Attribution license.
